# A Panel of Circulating Exosomal sncRNAs Associated With Lung Cancer Risk up to 10 Years in Advance

**DOI:** 10.1002/advs.76729

**Published:** 2026-08-14

**Authors:** Zhuokun Feng, Masaki Nasu, Ayman Abdul‐Ghani, Yu Chen, Shaoqiu Chen, Isam Mohd Ibrahim, Hanqiu Zhang, Lauren Higa, Zitong Gao, Hua Yang, Raphael Bueno, Loïc Le Marchand, Youping Deng

**Affiliations:** ^1^ Department of Quantitative Health Sciences John A. Burns School of Medicine University of Hawaii at Manoa Honolulu Hawaii USA; ^2^ Molecular Biosciences and Bioengineering Program College of Tropical Agriculture and Human Resources University of Hawaii at Manoa Honolulu Hawaii USA; ^3^ Genomics and Bioinformatics Shared Resource University of Hawaii Cancer Center Honolulu Hawaii USA; ^4^ Pali Momi Medical Center Honolulu Hawaii USA; ^5^ Division of Thoracic Surgery Brigham and Women's Hospital Boston Massachusetts USA; ^6^ Cancer Epidemiology Program University of Hawaii Cancer Center Honolulu Hawaii USA; ^7^ Population Sciences in the Pacific Program University of Hawai'i Cancer Center Honolulu Hawaii USA; ^8^ Pacific Center for Genome Research University of Hawaii Cancer Center Honolulu Hawaii USA

**Keywords:** exosome, machine learning, plasma, pre‐diagnosis biomarker, small non‐coding RNA

## Abstract

Current lung cancer screening fails to identify many individuals at risk beyond heavy smokers, highlighting the need for additional predictive biomarkers. This study profiles circulating exosomal small non‐coding RNAs (sncRNAs) in a prospective cohort of 202 smokers, including 68 incident lung cancer cases with up to 16 years of follow‐up. A structured 5 × 5 nested cross‐validation framework incorporating feature selection, classifier comparison, and outer‐loop evaluation identifies a panel of 31 sncRNA risk biomarkers. The optimized random forest model achieves an area under the curve (AUC) of 0.97 in outer‐fold testing (sensitivity = 0.93; specificity = 1.00). The derived risk score remains independently associated with incident lung cancer after adjustment for demographic and smoking variables (odds ratio [OR] = 13.19, 95% confidence interval [CI] 6.83–25.45) and is associated with shorter time‐to‐diagnosis in competing risks analysis (subdistribution hazard ratio [sHR] = 4.78, 95% CI 3.57–6.41). Associations are observed up to 10 years prior to diagnosis. Target gene analysis implicates pathways relevant to lung tumorigenesis, including PI3K‐Akt, p53, and MAPK signaling. These findings suggest that plasma exosomal sncRNAs may contribute to early lung cancer risk assessment and warrant further evaluation in independent pre‐diagnostic cohorts.

## Graphical abstract

Lung cancer is often diagnosed too late, and current screening overlooks many people at risk. In a long‐term study of smokers, a panel of small non‐coding RNAs carried in blood exosomes signals elevated lung cancer risk up to ten years before diagnosis, pointing toward a blood‐based tool for earlier risk detection.

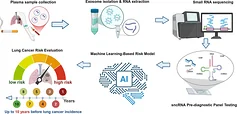

## 1 Introduction

Lung cancer remains the most prevalent malignancy and the leading cause of cancer‐related mortality all over the world, accounting for approximately 1.8 million deaths annually [1]. In the United States, projections for 2025 estimate around 226 650 new cases and 124 730 deaths, representing nearly 20% of all cancer‐related fatalities [1]. Patients’ prognosis is highly dependent on stage at diagnosis: the five‐year survival rate is approximately 65% for patients diagnosed at an early stage but declines sharply to about 9% for those identified at a late stage with distant metastasis [2]. Therefore, accurate risk evaluation and early screening are critical for improving the prognosis of lung cancer patients.

Current lung cancer screening guidelines, most notably from the U.S. Preventive Services Task Force (USPSTF) and the National Comprehensive Cancer Network (NCCN), recommend annual low‐dose computed tomography (LDCT) for high‐risk individuals. This population is generally defined as adults aged 50 to 80 years with a ≥20 pack‐year smoking history who currently smoke or have quit within the past 15 years [3, 4]. Individuals not meeting these specific criteria are not recommended for routine screening. However, while this risk stratification framework is a major clinical advance, it faces significant limitations. Firstly, its near‐exclusive reliance on heavy smoking history means that a growing population of at‐risk individuals, including never‐smokers and those with lighter smoking histories [5, 6], is entirely overlooked. Secondly, the dichotomous, “one‐size‐fits‐all” nature of these criteria fails to capture the true continuum of lung cancer risk. This rigid model does not account for the multifaceted interplay of other critical risk factors, such as family history, race and ethnicity, COPD, and occupational exposures, which can substantially elevate an individual's risk profile even if they fall outside the strict smoking criteria. These limitations highlight an urgent need for more precise and comprehensive risk prediction models to optimize the selection of candidates for lung cancer screening.

While current risk prediction models are founded on established risk factors like age and smoking history, their resolution is limited, particularly for individuals outside conventional high‐risk categories [7, 8, 9]. Tumorigenesis is a progressive process, and research confirms that the underlying molecular alterations can be detected in the bloodstream years before a tumor becomes clinically apparent [10, 11, 12]. Among a group of promising candidates for capturing these early signals are circulating small non‐coding RNAs (sncRNAs). Various classes—including miRNAs, piRNAs, and tRNA‐derived fragments—have been established as early diagnostic biomarkers [13, 14, 15]. For pre‐diagnostic signatures, specific panels of serum sncRNAs have been shown to identify individuals who will later develop lung cancer up to eight years before a clinical diagnosis [10, 12]. These findings collectively underscore a compelling opportunity to leverage a multi‐class sncRNA signature from liquid biopsies. Integrating such molecular data with traditional risk factors holds the potential to significantly enhance predictive accuracy [16, 17], facilitating a more timely, accurate, and personalized assessment of lung cancer risk.

Among the sources of circulating sncRNAs, exosomes have emerged as particularly valuable targets for liquid biopsy due to their important role in intercellular communication during cancer development [18]. As these extracellular vesicles transfer molecular cargo between tumor cells and the host microenvironment, their sncRNA content reflects the evolving molecular landscape throughout tumorigenesis. Exosomes are superior targets of liquid biopsy in cancer screening for several reasons: their membranes protect RNA from degradation, ensuring stability; they concentrate cell‐specific molecules, providing signal enrichment of certain diseases; and their isolation filters out plasma‐derived background noise and contaminants, ensuring purity [19, 20]. These features collectively yield a more reproducible and biologically relevant signal for clinical applications. Despite this clear potential, most exosome‐based biomarker studies have been conducted in the diagnostic setting using samples collected at or after clinical presentation [21, 22]. In contrast, investigations into the utility of exosomal sncRNAs as predictive biomarkers in pre‐diagnostic contexts remain scarce, largely due to the challenges of recruiting prospective cohorts and the limited availability of longitudinal samples.

In summary, advancing lung cancer risk prediction beyond traditional risk factors is a clinical imperative, holding the potential to fundamentally shift patient management towards proactive, early intervention. Despite this need, the specific role of exosomal sncRNAs as predictive biomarkers in the pre‐diagnostic window represents a significant unexplored area of research. Therefore, this study aimed to develop and validate a set of exosomal sncRNA predictive signatures to address the critical knowledge gap, with a final goal of creating a more accurate tool for early risk stratification and timely clinical intervention.

In this study, we recruited a longitudinal cohort of smokers to investigate alterations in circulating exosomal sncRNAs years before the clinical diagnosis of lung cancer. Our approach is distinguished by several key innovations. First, to overcome the challenges of high‐dimensional omics data within a limited‐size prospective cohort, we implemented a rigorous nested cross‐validation framework. This strategy not only ensures robust feature selection and model evaluation but also systematically identifies the optimal machine learning algorithm for the dataset. Specifically, our framework compared eight widely used algorithms to overcome the limitations of arbitrary algorithm selection, including logistic regression, penalized regression, random forest, support vector machine, gradient boosting, k‐nearest neighbors, decision tree, and neural network. Furthermore, moving beyond the conventional focus on miRNAs, our study establishes a comprehensive biomarker panel incorporating multiple sncRNA species (miRNAs, piRNAs, tiRNAs, and tRFs) to provide a more holistic view of the pre‐cancerous molecular landscape. Finally, moving beyond conventional models, our study establishes the biomarker's predictive validity under a more stringent competing risks framework, uniquely characterizing both its non‐linear dose‐response relationship with cancer incidence and the dynamic evolution of its predictive signal strength across short‐ and long‐term pre‐diagnostic windows. In a word, these contributions provide novel evidence for the value of circulating exosomal sncRNAs as pre‐diagnostic biomarkers.

## 2 Materials and Methods

### 2.1 Study Design Overview

This study proceeded in three primary stages, including 6 main steps (Figure 1). In the first stage of biomarker discovery, we profiled four classes of plasma exosomal sncRNAs (miRNAs, piRNAs, tiRNAs, tRFs) from 202 smokers in the UHCC cohort (68 incident lung cancer cases). Using a nested 5 × 5 cross‐validation framework that integrated differential expression, pruning, and eight machine‐learning classifiers, we identified a 31‐sncRNA panel and derived an individualized risk score. In the second stage, we evaluated the association between the derived risk score and lung cancer outcomes. Multivariable models assessed its independent predictive value after adjustment for demographic and smoking variables, and time‐to‐diagnosis analyses examined temporal risk gradients. Finally, we conducted functional characterization of the identified sncRNA panel. Target gene prediction and pathway enrichment analyses were performed to explore the biological relevance of the signature.

**FIGURE 1:**
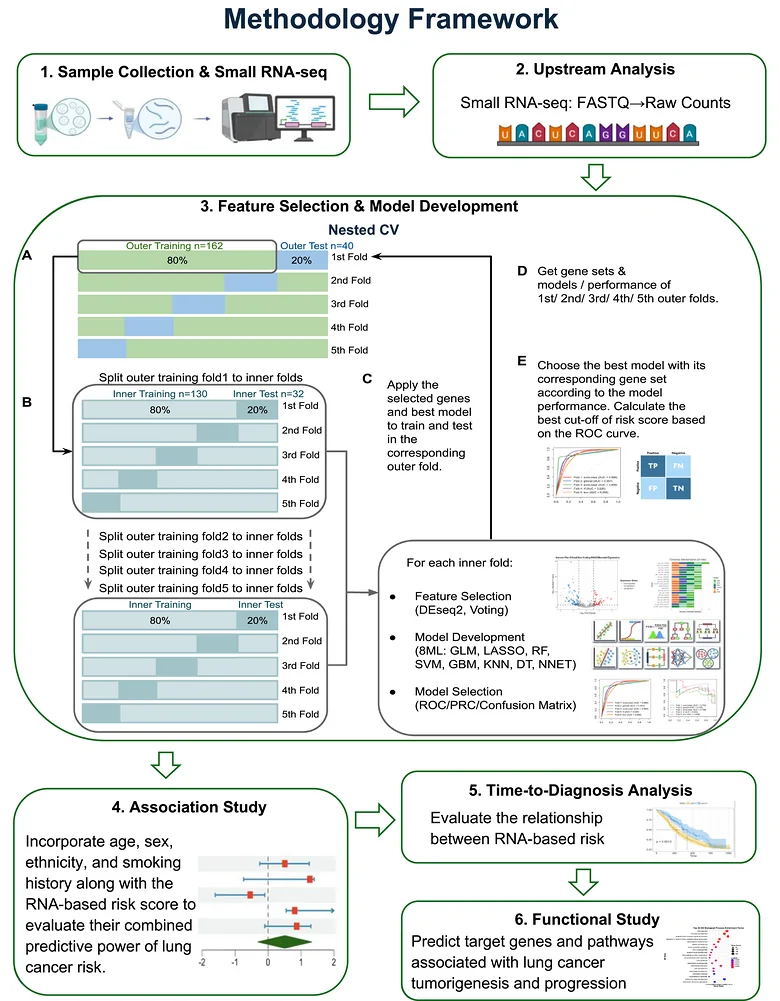
Methodology Framework.

### 2.2 Study Populations

The UHCC cohort was nested within a subset of current smokers from the Multiethnic Cohort Hawaii, primarily those enrolled in a nicotine metabolism investigation [23, 24]. Eligible participants were identified via baseline questionnaire data based on a smoking intensity of ≥10 cigarettes per day, no prior malignancy, and self‐reported Japanese, Caucasian, or Native Hawaiian ancestry. A total of 202 participants were included, comprising 68 individuals who subsequently developed lung cancer and 134 controls who remained cancer‐free, frequency‐matched (1:2) by self‐reported sex and ethnicity (Table 1). Peripheral blood samples were collected between 1994 and 2006 [25], in which all samples were obtained at baseline, preceding any cancer diagnosis or therapeutic intervention. Longitudinal follow‐up for cancer incidence was conducted through automated computer linkage with the Hawaii and California Tumor Registries, yielding a mean follow‐up duration of 13 years. For further validation, an independent cohort of 186 plasma samples was obtained from the Cooperative Human Tissue Network (CHTN), including 70 lung cancer cases and 116 cancer‐free controls. All samples were collected from participants diagnosed with early‐stage lung cancer (stages I and II) prior to the initiation of any clinical treatment (Data S1). Furthermore, to evaluate the biological persistence of the signature, publicly available data from The Cancer Genome Atlas (TCGA) for both LUAD and LUSC cohorts were incorporated. Following clinical data alignment, this dataset comprised 991 non‐small cell lung cancer (NSCLC) samples and 91 adjacent normal tissue samples.

**TABLE 1:** Demographic and clinical characteristics of the longitudinal UHCC cohort.

			Cancer‐free (n = 134)	Lung cancer (n = 68)	Total (n = 202)
			n (row%)	n (row%)	n (100%)
**Years Before Diagnosis**	≤ 5 years		4 (18.2)	18 (81.8)	22
	5‐10 years		15 (40.5)	22(59.5)	37
	10‐15 years		86 (76.1)	27(23.9)	113
	>15 years		29 (96.7)	1 (3.3)	30
**Sex**	Male		67 (66.3)	34 (33.7)	101
	Female		67 (66.3)	34 (33.7)	101
**Age**	Median		63	64.5	63.5
	Interquartile range		59 – 69.75	59 – 71	59 – 70
**Ethnicity**	European American		40 (66.7)	20 (33.3)	60
	Japanese American		42 (66.7)	21 (33.3)	63
	Native Hawaiian		52 (65.8)	27 (34.2)	79
**Smoking History**	Smoking Years	Median	43	44	43
		Interquartile range	38.25–47	38–49	38–47.75
	CPD	Light Smoker (CPD < 20)	23 (82.1)	5 (17.9)	28
		Moderate Smoker (20 ≤ CPD < 40)	72 (66.7)	36 (33.3)	108
		Heavy Smoker (CPD ≥ 40)	39 (59.1)	27 (40.9)	66
**Total**			134 (66.3)	68 (33.7)	202

### 2.3 Exosome Isolation and RNA Extraction

Extracellular vesicles (EVs) were isolated from 500 µL of plasma using a Capturem Extracellular Vesicle Isolation Kit (Mini, Takara Bio, Cat. No. 635741) according to the manufacturer's protocol. Purified EVs were eluted in 200 µL of the provided elution buffer. The identity and purity of the isolated EVs were subsequently confirmed. Transmission electron microscopy (TEM) revealed vesicles with the typical cup‐shaped morphology characteristic of exosomes (Figure S1a). Furthermore, Western blot analysis of EV lysate (70 µg protein) confirmed enrichment of the tetraspanin marker CD63 and the absence of the cellular contamination marker calnexin, which was present in the whole‐cell lysate control (36 µg protein) (Figure S1b). Total RNA was then extracted from the purified exosomes using the miRNeasy Serum/Plasma Kit (Cat. No. 217184; Qiagen, Germany), eluted in 16 µL of RNase‐free water, and stored at −80°C until further analysis.

### 2.4 Library Preparation and Small RNA Sequencing

Small RNA sequencing was performed on all 202 RNA samples by the Genomics and Bioinformatics Shared Resources (GBSR) at the University of Hawaii Cancer Center. Library preparation was conducted using the QIAseq miRNA Library Kit (Cat. No. 331502, Qiagen, Germany), according to the manufacturer's protocol. Sequencing was performed on the Illumina NextSeq 500 platform, targeting a depth of approximately 10 million reads per sample. This approach enabled a comprehensive characterization of the small RNA transcriptome in each plasma‐derived exosomal RNA sample.

### 2.5 Small RNA Sequencing Data Processing

Building upon the previously established small RNA‐seq preprocessing pipeline developed by our laboratory [26], we customized a workflow for the quantification of four sncRNA classes in this study: microRNAs (miRNAs), PIWI‐interacting RNAs (piRNAs), tRNA‐derived fragments (tRFs), and tRNA halves (tiRNAs). Quality control of all raw FASTQ files was first performed with *FastQC* (v0.11.9) to assess read quality, followed by adaptor trimming and filtering using *fastp* (v0.20.0). The processed reads were then aligned to the human reference genome GRCh38 (hg38) with *STAR* (v2.7.10). Gene feature annotation and quantification were carried out using *featureCounts* (v2.0.6) with custom Gene Transfer Format (GTF) annotation files specific to each sncRNA type. Specifically, miRNA annotation files were generated from the hg38 miRNA GFF file retrieved from miRBase (https://mirbase.org/). The piRNA reference was curated from the human hg38 piRNA BED file available through piRBase (http://bigdata.ibp.ac.cn/piRBase/index.php). Annotations for tRFs and tiRNAs were derived by converting hg19 tRF metadata obtained from MINTbase (https://cm.jefferson.edu/MINTbase/) to the hg38 genome build using the UCSC LiftOver tool (https://genome.ucsc.edu/cgi‐bin/hgLiftOver), followed by integration into a custom GTF file specific to hg38.

### 2.6 Nested Cross‐Validation Model for Biomarker Identification

We utilized the longitudinal cohort to identify pre‐diagnostic sncRNA biomarkers and develop the lung cancer risk prediction model. Given the limited sample size, particularly the inclusion of only 68 lung cancer patients, a nested cross‐validation (nested CV) strategy was employed to maximize data utilization and enhance the reliability of biomarker discovery. This integrative pipeline incorporated feature selection, normalization, model training, and testing based on the expression profiles of four sncRNA types: miRNA, piRNA, tiRNA, and tRF.

Raw counts were collected from the preprocessing pipeline and matched to sample diagnosis labels for each RNA category. To guarantee sufficient representation of cancer samples during validation, a two‐level nested CV design with five outer folds and five inner folds was implemented. The entire cohort was randomly stratified into five iterations of 80% outer training and 20% outer testing sets. Within each outer training set, five rounds of inner CV were conducted by further stratifying the data into 80% inner training and 20% inner testing subsets (Figure 1).

During each inner iteration, low‐expression features were filtered using the *filterByExpr* function in the package *edgeR* (v4.2.2), followed by differential expression analysis via *DESeq2* (v4.4.2). The top 50 RNAs per sncRNA class were selected based on absolute log2 fold change, using a significance threshold of p < 0.05. Transcripts per million (TPM) normalization was then applied using RNA‐specific lengths, followed by log2 transformation to reduce variance and normalize data distribution. Normalized features across RNA types were merged and subjected to correlation pruning to remove highly correlated (Pearson r > 0.9) and low‐variance RNAs, enhancing feature robustness and interpretability. The final feature set for each outer fold was determined by voting, retaining RNAs that appeared at least three times among the five inner folds.

To normalize and combine the expression data of selected biomarkers across different sncRNA classes, PCA was performed on the pruned features of each sncRNA class across outer folds to evaluate whether the standardized data (TPM‐log2) exhibited distinct clustering by RNA type (Figure S2). The PCA results showed largely overlapping distributions among sncRNA classes, suggesting the absence of strong class‐specific biases. Therefore, expression values of the selected features were further standardized using Z‐score normalization within each outer fold to place all selected RNAs on a comparable scale for subsequent model development. This unified approach ensured that features from different RNA classes contributed equally to the modeling process and that consistent procedures were applied across training and validation, thereby minimizing potential biases.

Before model training, the Synthetic Minority Over‐sampling Technique (SMOTE) in the package *DMwR* (v0.4.1) was applied within the training sets to address class imbalance. Then, eight ML algorithms were trained and evaluated on the inner test sets: logistic regression, penalized regression, random forest, support vector machine, gradient boosting, k‐nearest neighbors, decision tree, and neural network. To ensure numerical stability and avoid bias, certain inner folds were skipped under pre‐specified conditions: (i) insufficient informative features remained after filtering (e.g., <5 significant DE genes per RNA type, <5 total merged features, <2 features after variance or correlation pruning, or NA values in correlation matrices), which typically arose from small sample sizes, sparse expression profiles, or highly correlated features; (ii) data transformation or standardization inconsistencies prevented alignment between training and validation feature sets, even after zero‐filling missing genes, reflecting fold‐specific differences in selected features; or (iii) model training or probability‐based scoring failed, such as when a classifier could not output calibrated class probabilities for the cancer class, or when ROC computation failed due to degenerate predictions, often caused by limited sample sizes, imbalanced classes, or algorithm‐specific convergence issues. In such cases, each model failure led to omission of the affected model within a fold, whereas the entire fold was excluded only if no model produced valid probabilistic predictions and a computable ROC AUC. These safeguards reflect the realities of small‐sample, high‐dimensional data and ensure that unstable folds do not bias model selection. At last, the performance metrics from all folds were recorded, and the classifier achieving the highest average area under the receiver operating characteristic (ROC) curve (AUC) across inner folds was selected as the optimal model.

In each outer loop, the optimal model selected in the inner loops was retrained on the full outer training set using the corresponding selected RNA features. All preprocessing steps were confined to the outer training data to prevent information leakage. Expression values were re‐normalized (TPM followed by log_2_ transformation), and Z‐score parameters were estimated from the outer training set and applied to the held‐out outer test set. SMOTE oversampling was performed exclusively within the outer training data to address class imbalance.

The held‐out outer test set was not involved in feature selection, parameter estimation, or model tuning and served only for independent evaluation. Model performance across outer folds was assessed using ROC and precision‐recall (PRC) curve analyses. The final classifier and sncRNA feature set were determined based on comparative performance across outer test folds.

Risk scores were defined as the model‐predicted probabilities of lung cancer, with classification thresholds optimized using Youden's Index. Model performance was further evaluated using confusion matrices to calculate accuracy, sensitivity, and specificity. The risk score for each individual can be formally expressed as:

$$\mathrm{Risk}\;\mathrm{Scor}e_{i}=f_{\mathrm{model}}\;\left(z_{i}\right)$$

where:

-  $z_{i}=\left(z_{i1},\;z_{i2},\;z_{i3},\;\text{…},\;z_{in}\right)\;\;$ is the standardized feature vector for sample *i*, computed as:
$$z_{ij}=\frac{\mathrm{lo}g_{2}\left(\mathrm{TP}M_{ij}+1\right)-\mu_{j}}{\sigma_{j}}$$

with TPM_*ij*_being the TPM‐normalized expression of gene *j* in sample *i*, and μ_*j*_, σ_*j*_ representing the training set mean and standard deviation for gene *j*.

- *f_model_* ( · )is the probabilistic output function of the specific classifier, yielding the probability that sample *i* belongs to the positive class (lung cancer).

Below is a brief model‐specific interpretation of ​ *f_model_* for each of the eight classifiers:

1. Logistic Regression (GLM):
$$f_{\mathrm{GLM}}\left(z_{i}\right)=\frac{1}{1+e^{-\left(\beta_{0}+\sum_{j}\beta_{j}z_{ij}\right)}}$$

A linear combination of features passed through a sigmoid function to yield a probability.

2. Penalized Regression (LASSO / Elastic Net):

Same as GLM, but coefficients ​ β_*j*_ are estimated under regularization constraints to prevent overfitting:

$$\hat{\beta }=\arg\min_{\beta }\left\{-\ell \left(\beta \right)+\lambda \left[\alpha \;∥\beta {∥}_{1}+\left(1-\alpha \right)/2\;∥\beta {∥}_{2}^{2}\right]\right\}$$

3. Random Forest (RF):
$$f_{\mathrm{RF}}\left(z_{i}\right)=\frac{1}{T}\sum_{t=1}^{T}{\hat{p}}_{i}^{\left(t\right)}$$

The predicted class probabilities are averaged across *T* decision trees.

4. Support Vector Machine (SVM):
$$f_{\mathrm{SVM}}\left(z_{i}\right)=\frac{1}{1+e^{A\cdot d\left(z_{i}\right)+B}},\;\;d\left(z_{i}\right)=w^{⊤}\varphi \left(z_{i}\right)+b$$

The raw decision value is transformed into a probability using Platt scaling or similar methods.

5. Gradient Boosting Machine (GBM):
$$f_{\mathrm{GBM}}\left(z_{i}\right)=\frac{1}{1+e^{-F_{M}\left(z_{i}\right)}}$$

where *F_M_* is the output of the additive ensemble of ***M*** trees, converted to probability using a logistic link function.

6. k‐Nearest Neighbors (KNN):
$$f_{\mathrm{KNN}}\left(z_{i}\right)=\frac{1}{k}\sum_{j\in N_{k}\left(i\right)}I\left(y_{j}=1\right)$$

The proportion of lung cancer cases among the *k* nearest neighbors.

7. Decision Tree (rpart):
$$f_{\mathrm{Tree}}\left(z_{i}\right)=\hat{P}\left(y_{i}=1|\mathrm{lea}f_{i}\right)$$

The probability is estimated based on class distribution within the terminal node (leaf) to which **z**_*i*_ belongs.

8. Neural Network (NNET):
$$f_{\mathrm{NNET}}\left(z_{i}\right)=\sigma \left(w^{\left(2\right)}\cdot h\left(W^{\left(1\right)}z_{i}+b^{\left(1\right)}\right)+b^{\left(2\right)}\right)$$

A multi‐layer nonlinear transformation of input features, outputting a value between 0 and 1 via a sigmoid activation function σ(·).

This nested CV framework ensured robust feature selection and model generalizability, minimizing overfitting while accommodating the heterogeneity across RNA species. It enabled reproducible identification of multi‐class sncRNA biomarkers with strong potential for pre‐diagnostic lung cancer screening.

### 2.7 PCA for Discriminative Evaluation of sncRNAs Biomarkers

To evaluate the discriminative capacity of the selected RNA features, principal component analysis (PCA) was performed on expression data from the UHCC cohort using both the complete set of sncRNAs and the final sncRNA set. Raw count matrices for each sncRNA type (miRNA, piRNA, tiRNA, and tRF) were normalized by TPM and log2‐transformed to stabilize variance. The transformed matrices were then concatenated to generate combined feature matrices. Z‐score normalization was applied across all UHCC samples using the cohort‐wide mean and standard deviation. All samples were annotated with diagnostic labels standardized into two categories: “Lung Cancer” and “Cancer‐Free.” PCA was conducted using the *prcomp* function in the R package *stats* (v4.4.1), and the first two principal components (PC1 and PC2) were visualized with *ggplot2* (v3.5.2).

### 2.8 Tentative Validation in the CHTN Cohort

An independent set of 186 plasma samples from the CHTN cohort (Data S1) was analyzed to evaluate the signature's behavior in established clinical cases. Given the inherent differences in collection timing and cohort composition, this analysis was performed as an exploratory assessment.

Two normalization strategies were evaluated prior to analysis: (i) Z‐score transformation using the mean and standard deviation derived from the UHCC training set, and (ii) Z‐score transformation calculated within each dataset independently. Principal component analysis (PCA) was performed for both UHCC and CHTN cohorts (Figure S3). The latter approach was adopted to mitigate potential batch effects while preserving the internal structure of the CHTN data.

Three diagnostic contrasts were examined (Figure S4): (i) cancer vs. cancer‐free (benign and healthy combined), (ii) healthy vs. tumor (benign and cancer combined), and (iii) cancer vs. healthy. RNA expression counts (miRNA, piRNA, tiRNA, and tRF) were normalized to TPM with log_2_ transformation and merged by RNA class, followed by within‐cohort Z‐score standardization. The model trained on the UHCC cohort was applied to the standardized CHTN data to generate risk scores. Performance was assessed using receiver operating characteristic (ROC) and precision‐recall (PRC) analyses with the pROC (v1.18.5) and PRROC (v1.4) packages. AUC and AUPRC values with 95% confidence intervals were estimated via bootstrapping, and optimal thresholds were determined using Youden's Index.

### 2.9 Directional Consistency Analysis across Cohorts

To assess the biological persistence of the signature, we compared the direction of marker dysregulation in the UHCC cohort with that in the CHTN plasma cohort and TCGA lung tumor tissues (LUAD and LUSC). Raw counts for each dataset were separated by sncRNA class (miRNA, piRNA, tiRNA, and tRF) and converted to reads per million using all detected features within a class as the library size. We chose reads per million over model estimators to ensure comparability with curated tissue databases and because median‐of‐ratio estimates proved unstable for heavily dysregulated miRNAs in tumor tissues. The log2 fold change for each marker was then calculated as the log2 ratio of the mean class‐specific reads per million between groups (cancer vs. cancer‐free in UHCC, cancer vs. healthy in CHTN, and tumor vs. adjacent normal in TCGA), adding a pseudocount of one to each group mean.

Markers undetectable in a specific cohort were excluded, leaving 28 markers for the UHCC and CHTN comparison (excluding miR‐191‐5p, miR‐24‐3p, and miR‐3184‐3p) and all 31 markers for the UHCC and TCGA comparison. Individual marker significance was derived using a Wilcoxon rank‐sum test with Benjamini‐Hochberg adjustment strictly for reference, as this analysis relies on fold change direction (Data S5 and S6). To evaluate directional consistency between the UHCC and CHTN cohorts, markers were grouped into quadrants representing concordant upregulation or downregulation. Overall agreement was evaluated using a one‐tailed binomial test against a null concordance probability of 0.5, reported alongside the Spearman correlation for paired fold changes. The scatter plot was generated using R and the *ggplot2* package (Figure S5).

### 2.10 Model Sensitivity Analysis

To evaluate the potential influence of technical variance (e.g., fluctuations in exosome yield and sncRNA concentration) on the predictive model, we performed a multivariate Beta regression analysis. This approach was specifically designed to verify whether the pre‐diagnostic biomarker signals remained robust despite the potential degradation of biological components during long‐term sample storage.

Given that the sncRNA risk score is a continuous probability constrained within the interval [0, 1], the Beta distribution was employed as the link function. In this model, the risk score served as the dependent variable, while disease status and sequencing depth (categorized into “High” and “Low” subgroups by the median) were incorporated as independent covariates (Figure 3). The analysis, implemented via the *betareg* package in R (v4.2.0), estimated regression coefficients (β) on the logit scale and utilized Pseudo R2 to assess the variance explained.

By demonstrating a consistent association between the risk score and disease status across the 16‐year pre‐diagnostic cohort while adjusting for technical library‐size effects, we validated the stability of the molecular signature against potential time‐dependent sample attrition.

### 2.11 Out‐of‐Fold (OOF) Risk Score Calculation

The OOF risk scores were generated from the optimal sncRNA signatures and classifiers identified in each outer fold of the nested CV framework during biomarker discovery. In each outer fold, the corresponding feature set and model type were fixed based on the nested CV results. Raw counts in the outer training subset were normalized into TPM values, log_2_‐transformed, and standardized by z‐score normalization using the training set‐specific mean and standard deviation, consistent with the preprocessing applied during biomarker identification. The selected classifier was then trained on each balanced outer training subset after SMOTE oversampling and subsequently applied to its corresponding outer validation set to compute risk scores. The risk scores from all five outer validation folds were concatenated to obtain the complete OOF predictions for all subjects. In this way, we calculated an OOF risk score for each outer fold‐specific optimal model and feature set for subsequent analyses. This procedure prevented data leakage, ensured comparability across folds, and provided unbiased risk scores for downstream evaluations.

### 2.12 Association Study

To assess whether the sncRNA‐derived risk score offers predictive value beyond established lung cancer risk factors, we performed logistic regression analyses using data from the entire UHCC cohort. The diagnostic outcome variable was modeled via the *stats* (v4.4.1) package as a function of clinical and demographic covariates, including age group, sex, ethnicity, smoking intensity (cigarettes per day), smoking duration, and the standardized OOF risk score of the final model and sncRNA signatures. Univariable logistic regression analyses were first performed to examine the individual association of each predictor with lung cancer diagnosis. A multivariable logistic regression model was then fitted to evaluate the independent effect of the risk score while adjusting for all other covariates. To enhance interpretability, categorical predictors such as age group and smoking intensity were relabeled with descriptive factor levels. Odds ratios (ORs) and 95% CIs were calculated using the *autoReg* (v0.3.4) package in R.

### 2.13 Time‐to‐Diagnosis Analysis

To evaluate the pre‐diagnostic value of the risk score over time and while accounting for competing events during follow‐up, we first applied the Fine‐Gray competing risk model with time‐to‐diagnosis as the outcome variable. The time variable was defined as years from study enrollment to lung cancer diagnosis, censoring, or a competing event (death from causes other than lung cancer). The model was fitted using the *cmprsk* R package (v2.2.12), with covariates expanded into dummy variables. Subdistribution hazard ratios (sHRs) with 95% CIs were estimated for the continuous OOF risk score of the final model and other covariates.

To further characterize the dose‐response relationship between the risk score and time‐to‐diagnosis, we fitted the Fine‐Gray model with a restricted cubic spline (RCS) term for the OOF risk score. sHRs across the score distribution were estimated relative to the reference (score = 0), with 95% CIs derived from the model variance‐covariance matrix. Nonlinearity was tested using a Wald test of higher‐order spline terms.

For supplementary visualization, Kaplan‐Meier curves were generated by tertiles of the OOF risk score using the *survival* (v3.6.4) and *survminer* (v0.5.0) packages, which were used to illustrate the relationship between the predicted risk score and time‐to‐diagnosis.

To assess time‐window‐specific discrimination, we performed a landmark analysis across prespecified follow‐up windows of 0–5, 5–10, and 10–15 years. For each window, cases were participants diagnosed with lung cancer strictly within the interval; controls were participants who were at risk at the window start and remained free of lung cancer through the window end; participants who experienced competing events within the window were excluded from the analysis. The evaluation included not only the OOF risk scores derived from the final model and its sncRNA signature, but also the OOF risk scores from all five outer folds of the nested CV procedure, each corresponding to the optimal classifier and feature set identified within that fold. This design enabled a comprehensive assessment of performance and fold‐to‐fold variability. For each window and fold‐specific score, we computed ROC AUC using *pROC* (v1.18.5) and AUPRC using *PRROC* (v1.4). To ensure robust estimation of performance under imbalanced class distributions, we applied class‐stratified bootstrap resampling (R = 500), in which cases and controls were resampled with replacement within class. Given the limited sample size, particularly the small number of cases in certain windows, we did not perform prevalence‐adjusted PR analyses; instead, we report raw AUPRC values together with the observed window‐specific baseline precision (cases / [cases + controls]). For visualization, uncertainty was summarized by the across‐fold mean and standard deviation. For interpretability, ROC baselines were fixed at AUC = 0.5, and PRC baselines were set to the window‐specific prevalence.

Finally, the discriminative ability of the scaled risk score was additionally assessed by time‐dependent ROC analysis using the *timeROC* package in R (v0.4). AUC values were estimated at 3, 5, 10, and 15 years of follow‐up, calculated under the cumulative definition of cases (subjects diagnosed with lung cancer before or at the time point) and controls (subjects remaining cancer‐free beyond the time point).

### 2.14 Functional Study

To investigate the potential regulatory mechanisms of the identified sncRNAs, including miRNAs, piRNAs, tiRNAs, and tRFs, target genes were predicted using *IntaRNA* (v2.3.1). Predicted targets were filtered using an energy threshold of E ≤ ‐15 for downstream analyses. Gene Ontology (GO) enrichment analysis was performed on the filtered target genes for different sncRNA types in combination, covering the categories of Biological Process (BP), Cellular Component (CC), and Molecular Function (MF), using the *clusterProfiler* (v4.12.6) package in R. GO terms with adjusted p‐values < 0.05 (Benjamini‐Hochberg correction) were considered significantly enriched. From the combined GO‐BP results, cancer‐related biological processes were further extracted and visualized as bubble plots.

KEGG pathway enrichment analysis was conducted on the same gene sets using *enrichplot* (v1.24.4). Significantly enriched pathways (p.adj < 0.05) were retained, and pathways closely associated with lung cancer development were highlighted, such as Non‐small cell lung cancer, Small cell lung cancer, PI3K‐Akt signaling pathway, p53 signaling pathway, etc.

Within these pathways, key oncogenes (e.g., NRAS, MYC, EGFR) targeted by multiple sncRNAs were identified. These oncogenes, together with their regulating sncRNAs and associated pathways, were integrated into a sncRNA‐gene‐pathway regulatory network constructed in *Cytoscape* (v3.10.3), illustrating potential cooperative or synergistic roles of different sncRNA classes in lung tumorigenesis.

### 2.15 Statistical Analysis

All analyses were performed in *R* (v4.4.x). A nested 5 × 5 cross‐validation framework ensured unbiased feature selection, tuning, and model evaluation. Features were filtered (*edgeR*), tested for differential expression (*DESeq2, p<0.05*), normalized (TPM‐log_2_‐Z), and balanced by *SMOTE*. Eight classifiers were compared, and the model with the highest mean ROC AUC was selected. Out‐of‐fold (OOF) risk scores were used for unbiased prediction and association testing. Logistic regression estimated adjusted odds ratios (ORs), and Fine‐Gray competing risk models yielded subdistribution hazard ratios (sHRs) for time‐to‐diagnosis, with nonlinearity examined via restricted cubic splines. Landmark and time‐dependent ROC analyses evaluated discrimination across follow‐up intervals, and GO/KEGG enrichment identified biological pathways (FDR<0.05).

## 3 Results

### 3.1 Machine Learning‐Driven Biomarker Identification and Pre‐Diagnostic Model Construction

Using the nested CV framework, we simultaneously performed biomarker identification and model development for lung cancer pre‐diagnosis.

Within each outer training fold, differential expression analysis was first conducted in the corresponding inner folds to identify significantly dysregulated sncRNAs between lung cancer patients and cancer‐free individuals. This analysis was applied separately to each RNA class, including miRNAs, piRNAs, tiRNAs, and tRFs (Figure 2a). For each class, the top 50 RNAs ranked by absolute log_2_ fold change were selected, normalized, and merged into a combined expression matrix. To mitigate redundancy and multicollinearity, pairwise Pearson correlation filtering (|r| ≥ 0.9) was applied, and only representative features were retained for subsequent modeling. For instance, in inner fold 1 of outer fold 2, the feature set was reduced from 118 to 33 RNAs after correlation pruning (Figure 2b,c).

**FIGURE 2:**
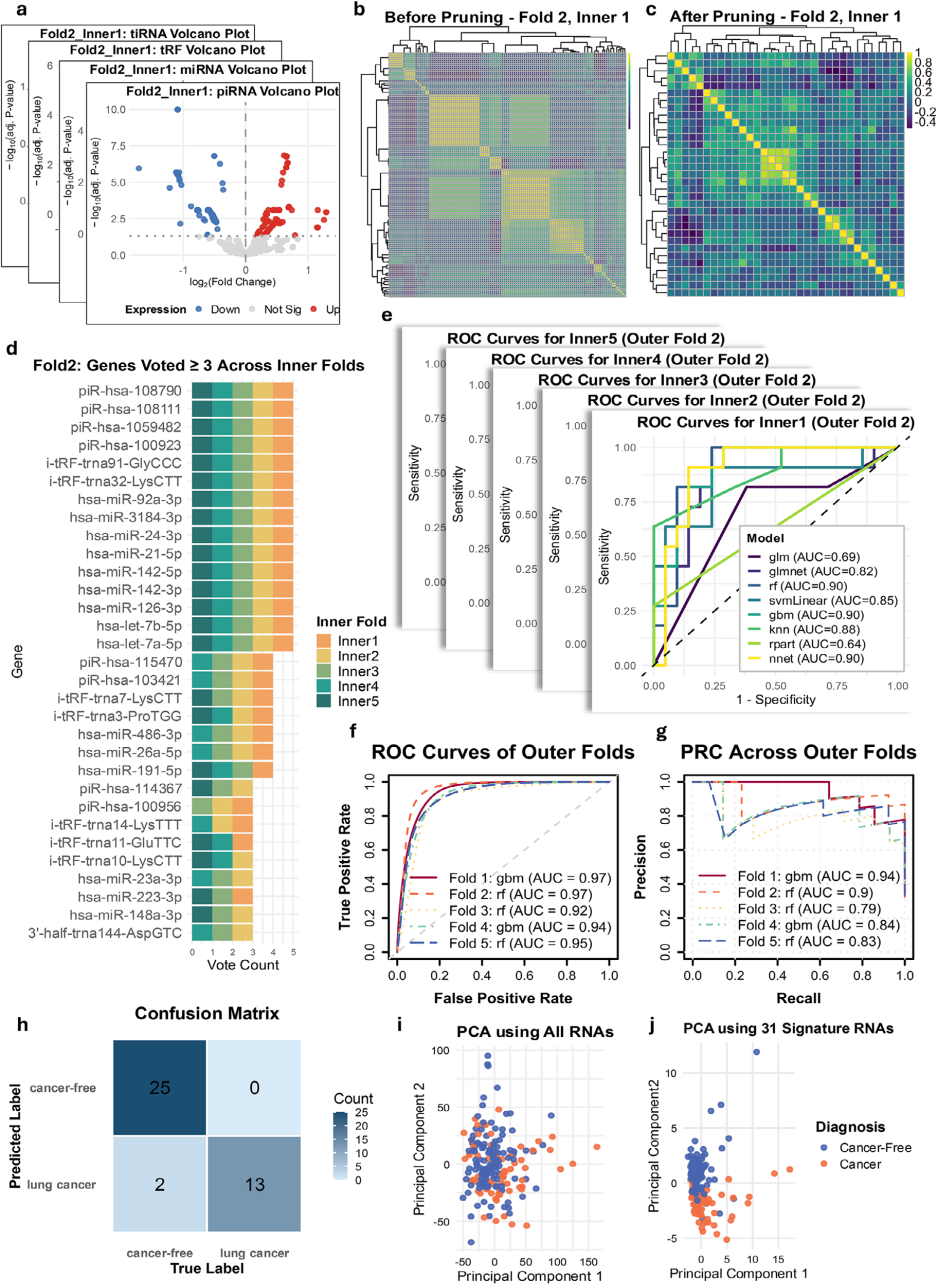
Nested cross‐validation workflow for feature selection, model training, and performance evaluation of sncRNA‐based lung cancer prediction. (a) Volcano plots from inner folds of outer fold 2 showing differential expression analysis of sncRNAs between lung cancer and cancer‐free samples across individual RNA classes (miRNAs, piRNAs, tRNAs, and tiRNAs). Significantly upregulated and downregulated RNAs are highlighted in red and blue, respectively. (b, c) Correlation heatmaps of the top‐ranked RNAs in inner fold 1 of outer fold 2 before and after correlation pruning. Highly correlated features (|r| ≥ 0.9) were removed, yielding a reduced and non‐redundant feature set (118 to 33 RNAs). (d) Heatmap of sncRNAs features repeatedly selected across inner folds of outer fold 2. RNAs with vote counts ≥3 were retained as the final feature set, yielding 31 pre‐diagnostic biomarker candidates. (e) ROC curves of eight machine learning algorithms tested in the inner folds of outer fold 2. Random forest (RF) achieved the highest mean AUC and was selected as the optimal classifier for this fold. (f) ROC curves across outer folds, showing robust classification performance with AUC values. (g) PRC across outer folds, demonstrating high precision and recall. (h) Confusion matrix of the final RF model in the outer test sets. (i, j) PCA plots of the UHCC cohort using all sncRNAs (i) and the 31 selected signature RNAs (j). Only the signature‐based PCA achieved clear separation between cancer and cancer‐free individuals.

The pruned features across all five inner folds of a given outer fold were then aggregated through a voting procedure: only RNAs repeatedly selected in more than three inner folds were retained as the final feature set for that outer fold (Figure S6). For example, in outer fold 2, this process consistently identified 31 sncRNAs, which were designated as pre‐diagnostic biomarker candidates (Figure 2d).

To determine the optimal predictive model, eight ML algorithms were evaluated in the inner CV, including logistic regression (GLM), penalized regression (GLMNET), support vector machine (SVM), random forest (RF), gradient boosting machine (GBM), k‐nearest neighbors (KNN), decision tree (RPART), and neural network (NNET). For each algorithm, fivefold CV was performed on the inner training data, and the average AUC across validation folds was used as the selection criterion. The model with the highest mean AUC in the given outer fold was chosen as the best classifier (Figure S7). In this step, we found that some algorithms were not successfully tested in certain inner folds. This occurred mainly when too few features remained after filtering, when probability outputs were unavailable due to model constraints (e.g., algorithms became unstable in small training sets), or when the positive class was absent in a given fold, leading to probability outputs without the expected positive class column. In rare cases, resampling also led to unstable class distributions. Such situations are expected in small‐sample, high‐dimensional settings, where data sparsity and model requirements can vary across folds. However, these skipped cases do not bias the overall conclusions, as model selection relied on aggregated results across all valid folds. Taking outer fold 2 as an example, RF achieved the highest inner‐loop AUC and was thus selected as the candidate model for that fold (Figure 2e).

In each outer loop, the selected model and its voted feature set were retrained exclusively on the outer training data and evaluated on the held‐out outer test set, which served as an independent validation set within the nested framework. Predictive performance across outer folds was assessed using ROC and precision‐recall curve (PRC) analyses (Data S2). Random Forest (RF) was selected as the optimal classifier in three of the five outer folds. Among these, the RF model in outer fold 2 demonstrated the highest validation performance (AUC = 0.97; AUPRC = 0.90) (Figure 2f,g). Based on outer‐fold validation results, the RF classifier trained with the 31 consistently selected sncRNAs was designated as the final pre‐diagnostic model, comprising 15 miRNAs, 8 piRNAs, 1 tiRNA, and 7 tRFs (Figure 2d).

Risk scores were then computed for each sample in the outer test sets using the final model. The optimal cut‐off value 0.50 was determined by Youden's Index, yielding a sensitivity of 0.93 and a specificity of 1.00, corresponding to an overall accuracy of 0.95 (95% CI: 0.83‐0.99; Figure 2h). The model performance was significantly superior to random classification (P [Accuracy > No Information Rate] = 2.989e‐05).

To further visualize discriminative ability, PCA was performed on the UHCC cohort using both the full sncRNA set and the 31 selected RNAs (Figure 2i,j). Before feature selection, cancer and cancer‐free samples showed no clear separation. By contrast, PCA based on the 31‐biomarker signature revealed a distinct separation between cancer and cancer‐free groups along the first two principal components, suggesting that the identified signature captures meaningful pre‐diagnostic signals between the two groups.

### 3.2 Spatiotemporal Heterogeneity of the Identified 31 Pre‐Diagnostic Biomarkers Across Clinical Stages and Sample Types

A tentative validation was performed using the clinically diagnosed CHTN cohort (Figure S4). Predictive performance showed some attenuation compared to the pre‐diagnostic setting (AUC range 0.63–0.73). These results likely reflect a combination of increased biological heterogeneity in the clinical setting, where the refined early‐warning signals are integrated into a more complex, noise‐heavy landscape of advanced tumorigenesis. To further evaluate the biological persistence of these markers, we performed a Directional Consistency Analysis comparing the Log_2_ fold changes between the UHCC (pre‐diagnostic) and CHTN (clinical) cohorts (Data S5 and Figure S5). The analysis revealed significant directional consistency, with 71% (20/28) of the markers exhibiting up‐ or down‐regulation consistently across both cohorts (binomial test, *p* = 0.018). While the correlation of the absolute fold‐change magnitudes showed a positive trend (Spearman's *R* = 0.311), it did not reach statistical significance (*p* = 0.107). This partial discordance suggests that while the foundational molecular signature persists, certain sncRNAs may undergo functional re‐orientation or stage‐specific regulation as the disease progresses from a pre‐clinical state to an overt malignancy.

Furthermore, we investigated the expression profiles of the 31 identified biomarkers in lung cancer tissue samples using the TCGA cohort (Data S6). Interestingly, several well‐documented oncogenic and tumor‐suppressive markers exhibited the expected up‐ or down‐regulation in tissue, which is consistent with previous literature. For instance, classic oncogenes (such as hsa‐miR‐92a‐3p, hsa‐miR‐142‐3p, hsa‐miR‐21‐5p, hsa‐miR‐23a‐3p, and hsa‐miR‐191‐5p) were confirmed to be up‐regulated in tumor samples, whereas classic tumor suppressors (such as hsa‐let‐7a‐5p, hsa‐let‐7b‐5p, hsa‐miR‐486‐3p, and hsa‐miR‐126‐3p) exhibited the expected down‐regulation. This biological concordance indirectly underscores the reliability of our upstream quantification and case‐control comparison methodologies. However, their expression directions in the plasma exosome cohorts (UHCC and CHTN) were not always consistent with the tissue profiles. For example, classic oncogenes (hsa‐miR‐21‐5p, hsa‐miR‐23a‐3p, and hsa‐miR‐92a‐3p) were consistently up‐regulated in lung cancer samples, and tumor suppressors (hsa‐miR‐223‐3p and piR‐hsa‐100956) were consistently down‐regulated in cancer samples across all three cohorts. Conversely, the oncogene hsa‐miR‐142‐5p was up‐regulated in tumor tissue but down‐regulated in cancer samples of both blood cohorts. Similarly, classic tumor suppressors such as hsa‐let‐7a‐5p, hsa‐let‐7b‐5p, and hsa‐miR‐486‐3p were down‐regulated in tumor tissue but up‐regulated in the circulating blood samples. This compartment‐specific discordance suggests that certain circulating sncRNAs may undergo selective secretion or active sorting mechanisms into the bloodstream exosome, rather than merely reflecting passive release from the primary tumor.

### 3.3 The Pre‐Diagnostic Risk Score is Driven by Disease Status and Independent of Technical Variance

To confirm that the 31‐sncRNA biomarker panel reflects intrinsic biological shifts rather than technical artifacts, we evaluated the potential impact of exosome abundance using total mapped reads as a proxy. Multivariate Beta regression demonstrated that sncRNA‐derived risk score distributions remained remarkably consistent regardless of sequencing depth (Figure 3a). Notably, disease status was identified as the sole significant driver of the biomarker (β = 3.781, *p* < 2 × 10^−16^), whereas sequencing depth exerted no independent effect (β = −0.012, *p* = 0.935; Figure 3b). The high Pseudo R^2^ (0.725) further confirms that the risk score's intensification as diagnosis approaches results from evolving sncRNA expression profiles rather than fluctuations in exosomal yield. These findings underscore the longitudinal stability of the pre‐diagnostic risk score throughout the follow‐up, providing a robust foundation for subsequent association analyses.

**FIGURE 3:**
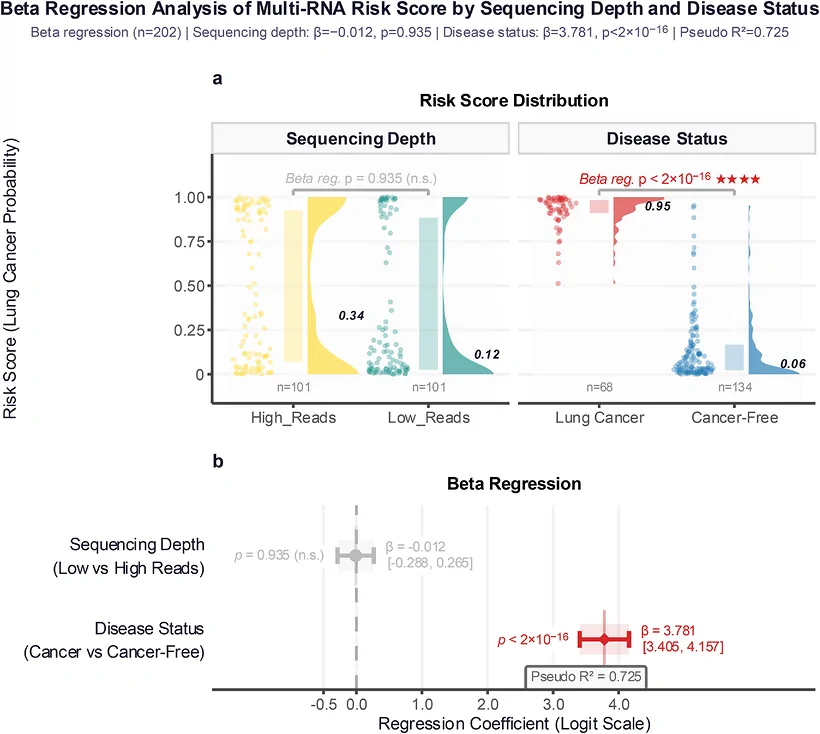
Robustness of the 31‐sncRNA Risk Score against sequencing depth and disease status (a) Raincloud plots showing the distribution of multi‐RNA risk scores stratified by sequencing depth (High Reads vs. Low Reads, left) and disease status (Lung Cancer vs. Cancer‐Free, right). Each panel displays individual data points, kernel density estimates, and boxplots (median ± IQR). Group medians are annotated. Beta regression p‐values are shown above brackets; n.s., not significant. (b) Forest plot of beta regression coefficients from a multivariable model including both sequencing depth and disease status (n = 202). Points represent coefficient estimates; horizontal lines indicate 95% confidence intervals. Diamond shape denotes statistical significance. Pseudo R^2^ = 0.725 indicates strong model fit.

### 3.4 The Pre‐Diagnostic Risk Score is an Independent Predictor for Lung Cancer

To evaluate the independent predictive value of the sncRNA‐derived OOF risk score for lung cancer prediction, both univariable and multivariable logistic regression analyses were performed on the UHCC cohort using epidemiological covariates (Figure 4a and Table 2). In the univariable analyses, heavy smoking status (defined by CPD criteria as CPD ≥ 40) showed a significant association with lung cancer (OR = 3.18, 95% CI: 1.08–9.42, p = 0.036), while other factors such as age, sex, ethnicity, smoking duration, and light/moderate smoking intensity were not statistically significant (all p > 0.05). Importantly, the standardized risk score demonstrated a strong and significant association with lung cancer diagnosis, with an odds ratio of 10.52 (95% CI: 5.91–18.71, p < 0.001) in the univariable model.

**FIGURE 4:**
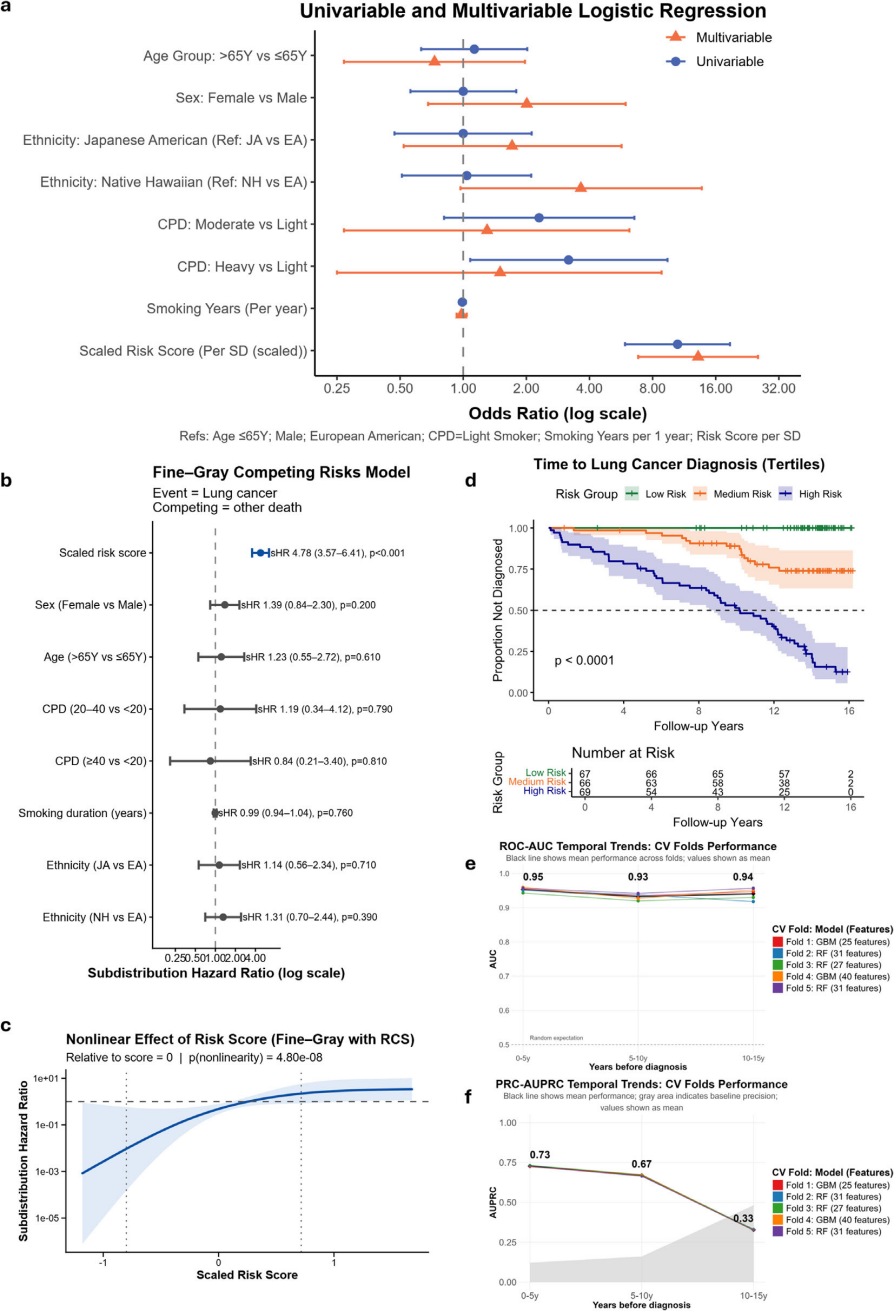
Independent predictive value and temporal performance of the sncRNA‐derived risk score for lung cancer. (a) Forest plot of univariable (blue circles) and multivariable (orange triangles) logistic regression analyses of clinical covariates and the sncRNA‐derived OOF risk score. Odds ratios are shown on a log scale with 95% confidence intervals. (b) Forest plot from a Fine‐Gray competing risks model showing that the sncRNA‐derived out‐of‐fold (OOF) risk score was strongly associated with lung cancer incidence (sHR = 4.78, 95% CI: 3.57–6.41, p < 0.001), while demographic and smoking‐related covariates were not significant. (c) RCS analysis showing a significant nonlinear effect of the risk score on lung cancer incidence, with risk escalating sharply at low values and plateauing at higher levels. (d) Kaplan‐Meier curves stratified by tertiles of the risk score, showing significantly earlier diagnoses in the high‐risk group (log‐rank p < 0.0001). (e) ROC‐AUC trends across time windows, showing consistently high discrimination with mean AUC values of 0.93–0.95 up to 15 years before diagnosis. (f) PRC‐AUPRC trends across time windows, with highest precision in the 0‐5‐year window (0.73) and a marked decline at longer pre‐diagnostic intervals (0.33 at 10–15 years), indicating weaker biomarker signal further from diagnosis.

**TABLE 2:** Logistic regression analyses of clinical variables and the sncRNA‐derived risk score.

Dependent: Diagnosis		Cancer‐Free (N = 134)	Lung Cancer (N = 68)	OR (Univariable)	OR (Multivariable)
**Age Group**	≤65Y	71 (53%)	34 (50%)		
	>65Y	63 (47%)	34 (50%)	1.13 (0.63–2.02), p = 0.688	0.73 (0.27–1.97), p = 0.540
**Sex**	Male	67 (50%)	34 (50%)		
	Female	67 (50%)	34 (50%)	1.00 (0.56–1.79), p = 1.000	2.01 (0.68–5.95), p = .207
**Ethnicity**	European American	40 (29.9%)	20 (29.4%)		
	Japanese American	42 (31.3%)	21 (30.9%)	1.00 (0.47–2.12), p = 1.000	1.71 (0.52–5.69), p = .380
	Native Hawaiian	52 (38.8%)	27 (39.7%)	1.04 (0.51–2.11), p = .917	3.64 (0.97–13.73), p = .056
**CPD**	Light Smoker	23 (17.2%)	5 (7.4%)		
	Moderate Smoker	72 (53.7%)	36 (52.9%)	2.30 (0.81–6.55), p = .119	1.30 (0.27–6.20), p = .741
	Heavy Smoker	39 (29.1%)	27 (39.7%)	3.18 (1.08–9.42), p = .036	1.50 (0.25–8.83), p = .655
**Smoking Years**	Mean ± SD	42.1 ± 7.7	41.7 ± 8.1	0.99 (0.96–1.03), p = .736	0.98 (0.93–1.04), p = .570
**Scaled Risk Score**	Mean ± SD	−0.5 ± 0.7	1.0 ± 0.6	10.52 (5.91–18.71), p<.001	13.19 (6.83–25.45), p<.001

Odds ratios (ORs) with 95% confidence intervals (CIs) and p‐values are shown; CPD: cigarettes per day; SD: standard deviation. Smoking status was defined based on cigarettes per day (CPD) as: light smoker (CPD < 20), moderate smoker (20 ≤ CPD < 40), and heavy smoker (CPD ≥ 40).

In the multivariable logistic regression, after adjusting for all other covariates, the risk score remained highly significant and independently predictive of lung cancer status (OR = 13.19, 95% CI: 6.83–25.45, p < 0.001). Notably, other traditional risk factors, including age group, sex, ethnicity, smoking intensity, and duration, were no longer statistically significant after adjustment (all p > 0.05), suggesting that the risk score can recognize critical predictive information not fully explained by these classic variables.

### 3.5 The Pre‐Diagnostic Risk Score Robustly Predicts Time to Lung Cancer Diagnosis Across Extended Follow‐Up

We evaluated the association between the sncRNA‐derived OOF risk score and time to lung cancer diagnosis using data from the UHCC cohort. In the Fine‐Gray competing risks model, the small RNA‐derived OOF risk score was strongly associated with lung cancer incidence after accounting for competing deaths (sHR = 4.78, 95% CI: 3.57–6.41, p < 0.001; Figure 4b and Table 3). None of the demographic or smoking‐related covariates, including sex, age, cigarette consumption, smoking duration, or ethnicity, showed significant associations in the multivariable model. These results suggest that higher risk scores were linked to a greater likelihood of earlier lung cancer occurrence during follow‐up, independent of demographic and smoking‐related factors.

**TABLE 3:** Fine‐Gray competing risks analysis of the sncRNA‐derived risk score and clinical covariates.

Variable	Sample Size (Lung Cancer/Censored/Competing)	sHR	95% CI	p value
Scaled risk score	68/88/46	4.78	3.57–6.41	<0.001
Sex (Female vs. Male)	34/47/20 vs. 34/41/26	1.39	0.84–2.30	0.20
Age (>65 vs. ≤65 years)	34/35/28 vs. 34/53/18	1.23	0.55–2.72	0.61
CPD (20–40 vs. <20)	36/50/22 vs. 5/16/7	1.19	0.34–4.12	0.79
CPD (≥40 vs. <20)	27/22/17 vs. 5/16/7	0.84	0.21–3.40	0.81
Smoking duration (years)	68/88/46	0.99	0.94–1.04	0.76
Ethnicity (JA vs. EA)	21/30/12 vs. 20/24/16	1.14	0.56–2.34	0.71
Ethnicity (NH vs. EA)	27/34/18 vs. 20/24/16	1.31	0.70–2.44	0.39

Subdistribution hazard ratios (sHRs) with 95% confidence intervals (CIs) and p‐values are shown. The “Sample Size (Lung Cancer / Censored / Competing)” column denotes the number of subjects in each covariate category who experienced: Lung Cancer (incident lung cancer diagnosis as the event of interest), Censored (no lung cancer diagnosis observed during follow‐up), and Competing (non‐lung cancer death occurring before lung cancer diagnosis, treated as a competing event in the Fine‐Gray model). CPD: cigarettes per day; EA: European American; JA: Japanese American; NH: Native Hawaiian.

RCS analysis of the Fine‐Gray model revealed a significant nonlinear association between the risk score and lung cancer incidence (p = 4.80 × 10^−8^). At the lower end of the score distribution, the sHR showed a sharp escalation, indicating that individuals with initially low scores were particularly sensitive to small increases in risk. In contrast, the curve plateaued in the mid‐to‐high range, suggesting that once individuals reached moderate risk levels, further increases caused limited additional hazard (Figure 4c).

Consistently, Kaplan‐Meier curves stratified by tertiles of the risk score demonstrated clear separation among low‐, medium‐, and high‐risk groups, with significantly earlier diagnoses observed in the high‐risk group (log‐rank p < 0.0001; Figure 4d).

In the landmark analyses, the sncRNA‐derived OOF risk scores showed divergent performance profiles when evaluated by ROC‐AUC versus PRC‐AUPRC (Figure 4e,f and Data S3 and S4). ROC‐AUC values remained uniformly high across all time windows, with mean AUCs of 0.93–0.95 across folds, indicating robust global case‐control discrimination even up to 15 years before diagnosis (Figure 4e). By contrast, PRC‐AUPRC provided a stricter assessment of ranking efficiency. Average precision was highest in the 0–5‐year window (0.73), decreased modestly in the 5–10‐year window (0.67), and dropped sharply in the 10–15‐year window (0.33), falling below the baseline precision defined by case prevalence (Figure 4f). This difference reflects the fact that ROC‐AUC captures overall ranking ability, which remained robust. In contrast, PRC‐AUPRC emphasizes the concentration of true cases among the highest‐scoring individuals, which declined notably as the pre‐diagnostic interval extended. The declining AUPRC at longer time before diagnosis indicates that the pre‐diagnostic biomarker signal is weaker at the earliest stages of carcinogenesis, making prediction more challenging further from the point of clinical detection. Additionally, the fold‐to‐fold results were highly consistent across all classifiers and sncRNA signatures (Data S3 and S4), indicating that these temporal trends represent a generalizable property of the risk scores rather than an artifact of any single model or feature set.

Supplementally, time‐dependent ROC analyses confirmed the robustness of the risk score across different follow‐up horizons. The score achieved AUCs of 0.83 at 3 years (n = 200; 12 cases, 188 controls), 0.83 at 5 years (n = 198; 18 cases, 180 controls), 0.86 at 10 years (n = 183; 40 cases, 143 controls), and 0.91 at 15 years (n = 97; 67 cases, 30 controls; Figure S8). These findings indicate that the sncRNA‐derived risk score provides meaningful discrimination as early as 3–5 years before clinical diagnosis, further confirming the result in the landmark analyses. The increase in AUC values observed at longer follow‐up primarily reflects the cumulative nature of cancer diagnosis and the reduction of individuals remaining cancer‐free over time.

### 3.6 The Pre‐Diagnostic Biomarker Convergently Regulates Key Oncogenic and Immune Pathways in Lung Cancer

To investigate the regulatory landscape of the identified 31 sncRNA biomarkers in lung cancer development, we utilized IntaRNA to predict the target genes of each sncRNA class. A total of 11,625 protein‐coding genes were identified as potential targets with statistically significant interaction probabilities (Data S7). Specifically, 3 939 genes were predicted to be regulated by miRNAs, 7 948 by piRNAs, 4 343 by tiRNAs, and 4 112 by tRFs, highlighting widespread post‐transcriptional regulation across the genome.

Functional enrichment analyses of the predicted targets were conducted to explore the biological implications of these regulatory interactions (Data S8 and S9). GO enrichment analysis of BP terms revealed that sncRNA targets were significantly involved in pathways relevant to tumor progression, immune modulation, and tissue development (Figure 5a). Notably, GO terms related to cell adhesion (e.g., positive regulation of cell adhesion, cell‐substrate adhesion, cell‐matrix adhesion) were among the top‐enriched categories, underscoring potential roles of sncRNAs in tumor invasion and metastasis. Apoptosis‐related processes, including the extrinsic apoptotic signaling pathway and neuron apoptotic process, were also highly enriched, reflecting the dysregulation of programmed cell death during tumorigenesis. Furthermore, the enriched positive regulation of the MAPK cascade highlights a strong association with oncogenic signaling in lung cancer. Enrichment in terms such as leukocyte proliferation, response to hypoxia, and extracellular matrix organization suggests that sncRNAs may influence immune cell dynamics and stromal remodeling within the lung tumor microenvironment. Developmental terms, including lung development and respiratory system development, further support the contribution of these targets to pathways relevant to lung cancer pathology.

**FIGURE 5:**
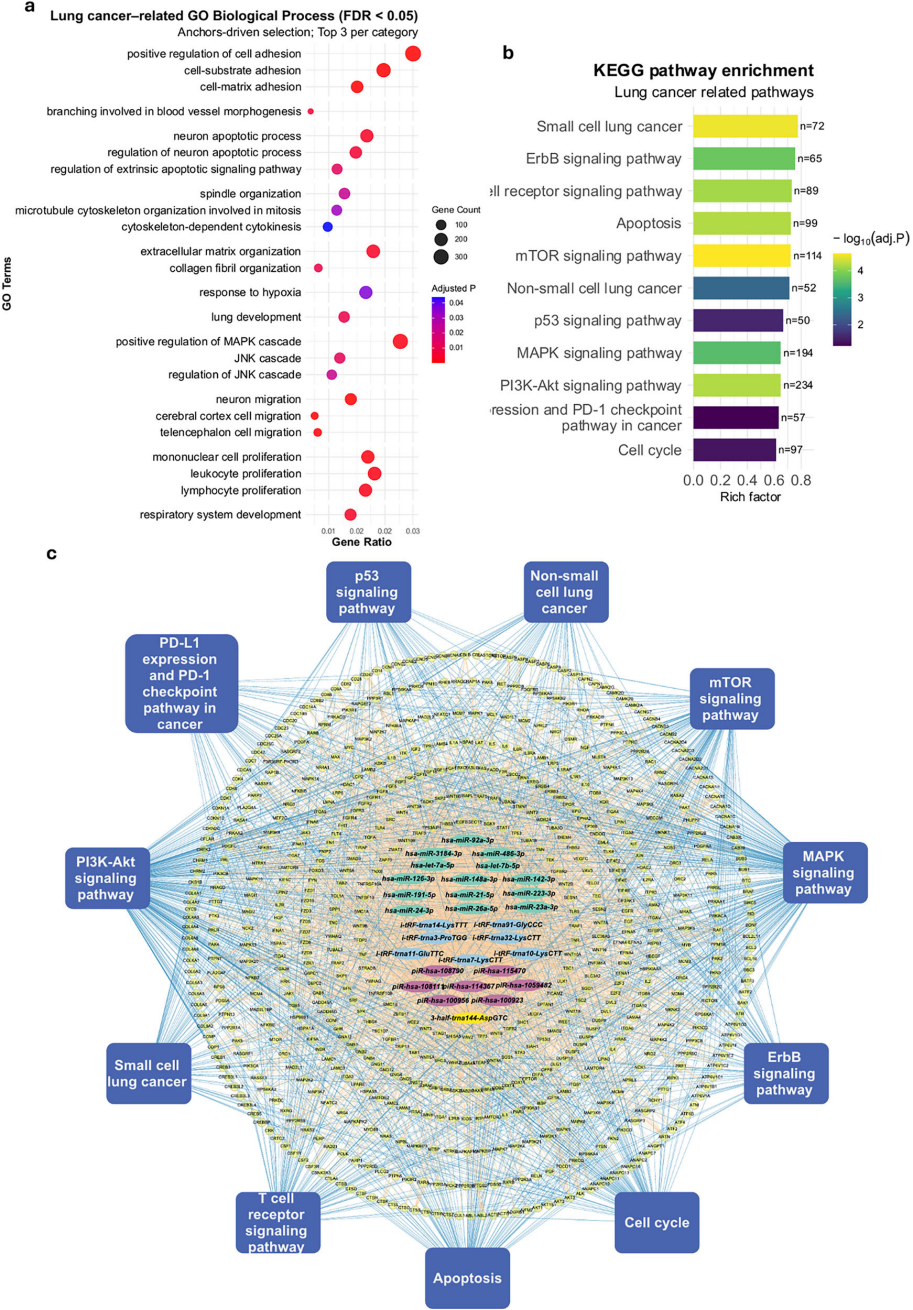
Functional characterization of predicted target genes of the 31 sncRNA biomarkers. (a) GO biological process enrichment (FDR < 0.05) of sncRNA target genes. (b) KEGG pathway enrichment showing significant enrichment of lung cancer‐associated and oncogenic pathways. (c) Integrated sncRNA‐gene‐pathway regulatory network linking the identified sncRNAs to key cancer‐related genes and signaling pathways, highlighting oncogenes and tumor suppressors regulated by multiple sncRNAs.

In the KEGG pathway enrichment analysis of the complete target gene set, we identified a panel of 11 lung cancer‐associated signaling pathways (Figure 5b). These included disease‐specific pathways, such as Non‐small cell lung cancer and Small cell lung cancer, as well as classical oncogenic pathways, such as PI3K‐Akt signaling, p53 signaling, MAPK signaling, ErbB signaling, and mTOR signaling. In addition, immune‐related pathways such as PD‐L1 expression and the PD‐1 checkpoint pathway in cancer and T cell receptor signaling were significantly enriched, indicating the involvement of sncRNA targets in tumor immune evasion mechanisms. Hallmark cancer pathways, including Cell cycle and Apoptosis, were also highlighted, consistent with known molecular hallmarks of cancer.

To further dissect the interplay between sncRNAs and cancer signaling, we constructed an integrated sncRNA‐gene‐pathway regulatory network (Figure 5c). Within this network, we identified several critical oncogenes and tumor suppressors, such as PIK3CD, AKT3, RASSF5, RARB, E2F2, ALK, RAF1, CASP9, LAMB2, and SOS1—as central nodes regulated by multiple sncRNAs. These genes are well‐established components of the non‐small cell lung cancer and small cell lung cancer signaling pathways and are known to modulate key processes, including proliferation, apoptosis, migration, and immune escape (Figure S9). Their coordinated regulation by diverse sncRNA classes suggests a potential convergent mechanism through which sncRNAs collectively influence lung cancer initiation and progression.

## 4 Discussion

In the evolving landscape of circulating biomarkers for early lung cancer detection, most established approaches focus on tumor‐derived signals, particularly cell‐free DNA (cfDNA) alterations such as mutations, methylation changes, and fragmentomic patterns. These assays have generally demonstrated high specificity but stage‐dependent sensitivity, with reduced detection rates in early‐stage disease. For example, in the CCGA independent validation of a targeted methylation multi‐cancer early detection test, specificity reached 99.5%, whereas overall sensitivity was 51.5%, including 16.8% for stage I cancers [27]. Lung‐focused fragmentomics approaches such as DELFI have similarly shown strong discrimination in post‐diagnostic cohorts (overall AUC ≈ 0.90), but more modest performance in stage I disease (AUC ≈ 0.76), and often rely on integration with clinical variables and CT imaging to balance sensitivity and specificity [28]. Evidence from truly pre‐diagnostic blood samples remains comparatively limited for cfDNA‐based assays, although longitudinal methylation analyses such as PanSeer reported high sensitivity (∼94.9%) at ∼96.1% specificity in samples collected 0–4 years prior to diagnosis [29], suggesting detectability within a preclinical window. In parallel, prospective studies have shown that circulating sncRNAs may exhibit measurable alterations years before clinical onset (e.g., AUC ≈ 0.76 up to 10 years pre‐diagnosis [10]), and have been evaluated as adjuncts to LDCT to reduce false positives [30]. Biologically, cfDNA primarily reflects tumor‐derived genetic material, whereas circulating sncRNAs may also capture systemic or host responses that precede substantial ctDNA shedding. This difference may arise because early tumorigenesis can induce alterations in the tumor microenvironment, immune signaling, and intercellular communication, leading to changes in exosomal RNA release even when tumor burden remains low. Recent multi‐modal approaches further indicate that integrating cfDNA methylation with protein and clinical features can enhance performance [31], supporting the potential value of complementary biomarker strategies in early detection.

Our study identified a panel of 31 plasma exosomal sncRNAs from a longitudinal cohort for lung cancer prediction, including 15 miRNAs, 8 piRNAs, 1 tiRNA, and 7 tRFs (Figure 2d). A random forest model was developed following comparison with seven commonly used machine learning algorithms (Figure 2f,g). The resulting sncRNA‐based risk score was associated with lung cancer incidence after adjustment for established risk factors, including age, sex, ethnicity, and smoking history (Figure 4a and Table 2). Time‐to‐diagnosis analysis showed that higher risk scores were generally observed among individuals diagnosed within shorter follow‐up intervals, with associations detectable up to 10 years prior to diagnosis (Figure 4b and Table 3). Finally, an sncRNA‐gene‐pathway regulatory network was constructed to explore potential biological pathways related to the identified biomarkers (Figure 5c).

Comparison with the published literature revealed that 16 of the identified biomarkers, mostly miRNAs, had been previously reported in lung cancer. The remaining 15, including 7 piRNAs and 8 tRF/tiRNAs, appear to represent novel or rarely reported candidates. Among the consistently reported biomarkers, miR‐142‐3p, miR‐21‐5p, miR‐24‐3p, hsa‐miR‐3184‐3p, miR‐92a‐3p, miR‐26a‐5p, and miR‐23a‐3p were upregulated in lung cancer patients. This is consistent with their recognized oncogenic roles, such as suppressing tumor suppressors including PTEN, SOX7, and PDCD4, or activating pro‐metastatic signaling pathways [32, 33, 34, 35, 36, 37, 38]. In contrast, miR‐126‐3p and miR‐223‐3p were downregulated, in line with their tumor‐suppressive functions in inhibiting PI3K/AKT and NLRP3‐mediated invasion [39, 40]. In addition, piR‐hsa‐100956 was significantly reduced in exosomes. This finding is consistent with recent liquid biopsy studies that highlight its diagnostic potential [15].

A subset of biomarkers, however, showed opposite trends compared with previous studies. For instance, let‐7a/b, miR‐486‐3p, and miR‐148a‐3p were upregulated in lung cancer samples of our pre‐diagnostic cohort, whereas they have been reported as downregulated tumor suppressors in lung tumor tissues [41, 42, 43]. Similarly, miR‐142‐5p and miR‐191‐5p were downregulated in our cancer samples, while prior studies described them as upregulated oncomiRs that promote immune evasion through the PTEN‐PD‐L1 axis or enhance cell proliferation [44, 45].

Several factors may explain these discrepancies, with the timing difference of sample collection being considered a critical reason. Our study analyzed pre‐diagnostic samples collected several years before clinical diagnosis. This design differs from most published studies, in which biomarkers were identified using samples obtained at or after lung cancer diagnosis. Accordingly, our identified biomarkers are more likely to reflect early biological alterations preceding clinical disease, potentially including processes such as immune modulation or metabolic reprogramming [46]. As the tumor progresses to a clinically diagnosed stage, the early predictive markers are potentially obscured by overwhelming signals related to high tumor burden and proliferation, demonstrating the critical difference between biomarkers optimized for prediction versus diagnosis [47, 48]. This distinction highlights an important difference between biomarkers optimized for long‐term risk prediction and those developed for clinical diagnosis [49, 50, 51]. This temporal specificity was directly corroborated in our tentative validation, where the comparison between the pre‐diagnostic UHCC cohort and the clinically diagnosed CHTN cohort revealed potential stage‐dependent shifts in biomarker profiles (Figures S4 and S5 and Data S5). In addition to this timing factor, differences in sample type are also relevant, since exosomal sncRNAs are subject to selective packaging and may not fully mirror tumor tissue expression as in other studies [52]. Our experimental findings robustly validate this hypothesis; upon examining the TCGA cohort, we observed that certain biomarkers exhibited opposite expression directions in lung cancer tissue compared to the circulating blood samples (Data S6). Finally, population heterogeneity, environmental exposures, and lifestyle factors may also contribute to variation in circulating sncRNA profiles [53]. In particular, our discovery cohort was primarily derived from residents of Hawaii and encompassed diverse ethnic backgrounds, including Native Hawaiian, European American, and Japanese American individuals. Moreover, all participants had various degrees of smoking history, which represents a major risk factor for lung cancer and a potential modifier of sncRNA expression [54, 55]. Overall, the identification of both concordant and discordant biomarkers highlights the complexity of circulating RNA biology in cancer development.

Traditional lung cancer risk factors such as age, sex, and smoking history are well established [1], but their predictive capacity remains modest. Large‐scale risk models based solely on these variables typically achieve moderate discrimination with AUCs in the range of 0.73–0.80 [56]. In our longitudinal cohort, heavy smoking status was associated with increased lung cancer risk (OR≈3.18) compared with light smokers, whereas other demographic and clinical variables were not statistically significant. By contrast, the sncRNA‐derived OOF risk score demonstrated a markedly stronger and independent association with lung cancer, showing an odds ratio of over 10 in univariable analysis and remaining highly significant after adjustment for all classical covariates (multivariable OR≈13.2). Importantly, once the sncRNA risk score was included in the model, none of the traditional predictors retained significance, indicating that the molecular signature captures critical information not encompassed by conventional variables. These findings are consistent with prior reports that integrating circulating sncRNA signatures into risk models significantly enhances diagnostic performance beyond smoking exposure and demographics, leading to improved sensitivity and specificity for lung cancer detection [57].

In the time‐to‐diagnosis analysis, the sncRNA‐derived risk score strongly predicted lung cancer earlier incidence (Fine‐Gray model sHR = 4.78, 95% CI: 3.57–6.41, *p* < 0.001), a result comparable to large‐scale microRNA studies such as BioMILD (HR ∼4.4) [58]. The RCS result within the Fine‐Gray framework further revealed a nonlinear dose‐response, indicating that the ORs from logistic regression represent the overall association between the sncRNA‐derived risk score and lung cancer risk across the entire cohort, while the nonlinearity and early‐occurrence patterns were more precisely captured in the time‐to‐diagnosis analyses. Additionally, the landmark analysis further reveals the dynamic temporal profile of a plasma exosomal sncRNA‐based risk score, providing basic insights into the molecular evolution of carcinogenesis up to 15 years before diagnosis. The declining AUPRC at longer pre‐diagnostic intervals indicates that while the score maintains robust global discrimination, its crucial ability to enrich for true cases among the highest‐risk individuals strengthens significantly as diagnosis approaches. This again underscores the dynamic and evolving nature of circulating biomarkers like exosomal RNAs [59, 60].

Our study's primary innovation lies in exploring the potential utility of circulating exosomal sncRNAs within the critical pre‐diagnostic window for lung cancer. We identified these molecules as promising pre‐diagnostic biomarkers, moving beyond their typical evaluation in diagnosed cohorts and, in doing so, addressing a critical gap by providing the first such predictive profile for the Hawaiian population. Furthermore, our identification of 15 novel predictive sncRNAs and the discovery that 6 known miRNA biomarkers exhibit opposite regulation compared to diagnosed cases decisively highlight the principle of stage‐specificity. This finding underscores the necessity of discovering and validating biomarkers specifically within a pre‐diagnostic context. Collectively, these innovations not only provide a new set of candidate biomarkers but also offer critical insights into the temporal dynamics of circulating RNAs during early carcinogenesis.

The pre‐diagnostic behavior of these biomarkers suggests their potential role in lung cancer screening. Because the panel was associated with lung cancer incidence up to a decade before diagnosis and stayed predictive after accounting for smoking and demographic factors, it could be developed into a blood‐based tool to help decide who is referred for LDCT. This may be particularly relevant for individuals at appreciable risk who currently fall outside eligibility criteria, including never‐smokers, lighter smokers, or those with other unmeasured clinical risk factors. Combined with existing risk models, the score could help target LDCT to those most likely to benefit, as in the miRNA‐based MILD and BioMILD trials [30, 58].

Translation into a clinical test would require transitioning from the genome‐wide sequencing used in this study for discovery to a streamlined, targeted small RNA sequencing assay restricted to the 31 markers. Genome‐wide profiling effectively captures relative abundance changes across cohorts. However, a targeted clinical panel, which is implemented via amplicon or hybrid‐capture, demands highly efficient capture of diverse RNA classes for absolute quantification. Because RNA classes like piRNAs (with 3′ 2′‐O‐methylation) and tRNA‐derived fragments (with modified bases) exhibit distinct biochemical properties, optimizing their uniform recovery in a targeted format is an important next step [61, 62]. This can be achieved by incorporating a modification‐tolerant reverse transcriptase or an AlkB‐based demethylation step into the integrated workflow [63, 64]. Such optimizations will ensure efficient, quantitative recovery across all four classes (miRNA, piRNA, tiRNA, and tRF). Because only a fixed panel is read, the depth needed per sample falls well below the roughly ten million reads used for discovery, allowing hundreds of barcoded samples to be pooled cost‐effectively into a single run.

Clinical use would then depend on standardization and throughput. The plasma collection and exosome isolation protocol would be fixed, while exogenous spike‐ins are introduced to monitor recovery rates and technical variations. The cohort‐based Z‐scoring used here cannot be applied to a single patient and would be replaced with fixed normalization based on spike‐ins and stable endogenous RNAs. The normalized data will then feed into this prebuilt machine learning model to calculate the risk score and perform classification with the predefined 0.50 threshold. Assay parameters such as limits of detection, precision, and inter‐laboratory concordance must be established under a CLIA or CAP regulatory framework [65, 66]. Exosome isolation is the main constraint on scalability but can be moved to automated magnetic‐bead capture [67], and the resulting end‐to‐end turnaround of two to three days is acceptable for long‐range risk assessment rather than acute care. The test should be inexpensive relative to LDCT and could prove cost‐effective by reducing scans and false‐positive results, although a formal cost‐effectiveness analysis and a possible smaller core panel remain to be addressed.

Despite the robustness of our findings, this study has several limitations that provide important context and direct future research. The primary limitation is the modest sample size of our longitudinal cohort. To mitigate this, we employed a nested cross‐validation framework to maximize data utilization. Meanwhile, to test the stability of its performance, we conducted a simulation‐based power analysis on the OOF score of the second outer fold, which was selected as the final model and feature set in our study. The model demonstrated exceptionally strong and stable discriminative ability (AUC = 0.93, 95% CI: 0.90–0.97, DeLong test). Given that the lower bound of the confidence interval was substantially higher than the threshold for clinical relevance (AUC > 0.7), our stratified bootstrap resampling (B = 2 000) unsurprisingly yielded approximately 100% empirical power. Nevertheless, the sample size still contributed to unstable performance of some models during algorithm comparison, wide 95% confidence intervals for key risk estimates, and precluded subgroup analyses based on lung cancer subtypes or clinical stages. Another limitation is the lack of an external pre‐diagnostic cohort for validation, reflecting the practical difficulty of obtaining longitudinal plasma samples collected years before cancer diagnosis. We performed an exploratory comparison in a clinically diagnosed CHTN cohort; however, performance was attenuated (AUC ∼0.7, Figure S4), likely due to biological differences between post‐diagnostic and pre‐diagnostic states. These observations highlight the need for validation in similarly designed pre‐diagnostic cohorts to better assess reproducibility and clinical relevance. Beyond sample limitation, the composition of our cohort presents further considerations; the universal history of smoking may lead to an overestimation of the sncRNA score's independent predictive strength, and the high cancer prevalence compared to the general population warrants caution when generalizing our findings. Finally, while our bioinformatic analysis provides a plausible regulatory network, these associations are correlational and require further mechanistic validation through multi‐omics integration and functional laboratory experiments to elucidate the precise molecular pathways.

The limitations of this study provide a clear plan for future research essential for the clinical translation of our findings. The foremost priority is to validate our 31‐sncRNA biomarker panel in a larger and ethnically diverse longitudinal cohort. Such studies are crucial to confirm its predictive accuracy, refine risk estimates to achieve narrower confidence intervals, and investigate its performance across different lung cancer histological subtypes and clinical stages. To assess the signature's true independent value, these validation efforts should include a substantial population of never‐smokers and employ a frequency‐matched nested case‐control design. Moreover, the approach of matching future cancer cases with controls based on critical confounders like age, sex, and detailed smoking history will rigorously test the biomarker's additive value beyond established risks and mitigate potential cohort‐specific biases. Finally, moving beyond the correlational findings of our pathway analysis, functional studies are needed to elucidate the biological mechanisms of these sncRNAs. Integrating multi‐omics data with in vitro and in vivo experiments will be critical to determine how these molecules mechanistically contribute to early carcinogenesis.

## Author Contributions

**Masaki Nasu**: methodology, data curation, investigation, visualization, validation. **Ayman Abdul‐Ghani**: data curation, methodology. **Zhuokun Feng**: writing – review and editing, conceptualization, methodology, formal analysis, validation, investigation, visualization, writing – original draft. **Raphael Bueno**: methodology, supervision. **Zitong Gao**: methodology, software. **Hua Yang**: project administration. **Yu Chen**: writing – review and editing, investigation. **Isam Mohd Ibrahim**: methodology, software. **Shaoqiu Chen**: methodology, software. **Youping Deng**: supervision, resources, methodology, funding acquisition, conceptualization. **Loïc Le Marchand**: methodology, formal analysis, data curation, resources, project administration. **Hanqiu Zhang**: investigation, validation. **Lauren Higa**: methodology, software.

## Funding

This work was supported by the National Institutes of Health grant R01CA230514, the National Institutes of Health grant R01CA223490, the National Institutes of Health grant U01CA164973, the National Institutes of Health grant P20GM103466, the National Institutes of Health grant P30CA071789, the National Institutes of Health grant P20GM139753, the National Institutes of Health grant U54GM138062, the National Institutes of Health grant U54HG013243, the National Institutes of Health grant T32DK137523, the National Institutes of Health grant 1UE5HG013826, the National Institutes of Health grant 3OT2OD032581‐01S5‐895, the National Institutes of Health grant 1OT2OD032581‐02‐PP90Y, the National Institutes of Health grant U54MD007601, the National Institutes of Health grant P20GM161995, the National Institutes of Health grant 1R01CA316647, the National Institutes of Health grant 1OT2OD032581‐02.

## Ethics Statement

Ethical acquisition and utilization of human blood samples, including all subsequent molecular analyses and clinical data integration for this study, were approved by the University of Hawaii Human Studies Program (Institutional Review Board, IRB) under protocol numbers 2022‐00819 and 2018‐00636.

## Consent

Informed consent was obtained from all participants prior to sample collection. As samples were procured through the CHTN, individual informed consent was obtained directly by CHTN in compliance with their institutional protocols and federal regulations.

## Conflicts of Interest

The authors declare no conflicts of interest.

## Supporting information




**Supporting File 1**: advs76729‐sup‐0001‐SuppMat.docx.

**Supporting File 2**: advs76729‐sup‐0002‐DataS1‐S9.zip.

## Data Availability

The demographic and clinical information for the UHCC cohort were derived from a subset of current smokers within the Hawaiʻi component of the Multiethnic Cohort (MEC), primarily participants enrolled in a nicotine metabolism study (PMID: 19029401), and are publicly available through *Cancer Epidemiology, Biomarkers & Prevention* Online. Demographic and clinical information for the CHTN samples are provided in Supplementary Data S1. Raw sequencing data for the plasma samples are available from the corresponding authors upon reasonable request. Additionally, small RNA sequencing profiles and clinical metadata for the TCGA‐LUAD and TCGA‐LUSC cohorts were downloaded from the official Genomic Data Commons (GDC) Data Portal (https://portal.gdc.cancer.gov/).
