# Supplementary material for: A Panel of Circulating Exosomal sncRNAs Associated With Lung Cancer Risk up to 10 Years in Advance
Source: Adv Sci (Weinh). 2026 Aug 14;13(57):e76729. doi: 10.1002/advs.76729 (PMC13474057; doi:10.1002/advs.76729)
Supplement: Supplementary file 1 [file ADVS-13-e76729-s001.docx]

**A Panel of Circulating Exosomal sncRNAs Associated with Lung Cancer Risk up to 10 Years in Advance**

**Authors:** Zhuokun Feng^1,2,3^, Masaki Nasu^1^, Ayman Abdul-Ghani^4^, Yu Chen^1^, Shaoqiu Chen^1^, Isam Mohd Ibrahim^1^, Hanqiu Zhang^1^, Lauren Higa^1^, Zitong Gao^1^, Hua Yang^1^, Raphael Bueno^5^, Loïc Le Marchand^6, 7*^, Youping Deng^1,3,8*^

**Affiliations:**

^1^Department of Quantitative Health Sciences, John A. Burns School of Medicine, University of Hawaii at Manoa; Honolulu, HI, USA, 96813.

^2^Molecular Biosciences and Bioengineering Program, College of Tropical Agriculture and Human Resources, University of Hawaii at Manoa; Honolulu, HI, USA, 96822.

^3^Genomics and Bioinformatics Shared Resource, University of Hawaii Cancer Center; Honolulu, HI, USA, 96813.

^4^Pali Momi Medical Center; Honolulu, HI, USA, 96701.

^5^Division of Thoracic Surgery, Brigham and Women's Hospital, 75 Francis St; Boston, MA, USA, 02115.

^6^Office of Public Health Studies, University of Hawaii at Manoa; Honolulu, HI, USA, 96813.

^7^Biomedical Sciences - Clinical Research Track and Cell and Molecular Biology Program, John A. Burns School of Medicine, University of Hawaii at Manoa; Honolulu, HI, USA, 96813.

^8^Pacific Center for Genome Research, University of Hawaii Cancer Center; Honolulu, HI, USA, 96813.

# List of Supplementary Materials

Figure S1 Transmission electron microscopy and Western blot of plasma-derived exosomes.

Figure S2 PCA projection of pruned sncRNA features by RNA type.

Figure S3 Comparison of normalization strategies for cross-cohort analysis.

Figure S4 Performance of the 31-sncRNA panel in the CHTN diagnosed cohort.

Figure S5 Directional Consistency and Correlation Analysis of the 31-sncRNA Signature Between Pre-diagnostic (UHCC) and Clinical (CHTN) Cohorts.

Figure S6 Outer fold feature sets derived from inner-fold voting.

Figure S7 ROC curves from inner folds of each outer fold for eight machine learning algorithms (GLM, GLMNET, SVM, RF, GBM, KNN, RPART, NNET).

Figure S8 Time-dependent ROC analysis of the sncRNA-derived risk score.

Figure S9 Integrated sncRNA-gene-pathway regulatory network in lung cancer.

[Data S1. CHTN sample information](https://drive.google.com/file/d/1TZhMtlMTvLb__VwmWT_xmxZ9Kc3nd9XE/view?usp=drive_link)

[Data S2. Results of nested cross-validation](https://drive.google.com/file/d/11vd8JZbDA4Kv81ci-kz6Hl5alC4vQzx_/view?usp=drive_link)

[Data S3. ROC-AUC result of landmark analysis](https://drive.google.com/file/d/1AErC5qpTzuCdfKky8HvmME8jAuHpIYiD/view?usp=drive_link)

[Data S4. PRC-AUPRC result of landmark analysis](https://drive.google.com/file/d/1JAHcyhJ3QypEOSCedwqFhbzZX7tNFTE1/view?usp=drive_link)

Data S5. Directional consistency analysis UHCC-CHTN

Data S6. Directional consistency analysis UHCC-TCGA

[Data S7. IntaRNA result](https://docs.google.com/spreadsheets/d/1x7gb34trjCfEaLgMIMdJQ5pipKUQBWcP/edit?usp=drive_link&ouid=118228466349625254909&rtpof=true&sd=true)

[Data S8. GO result](https://docs.google.com/spreadsheets/d/1x7gb34trjCfEaLgMIMdJQ5pipKUQBWcP/edit?usp=drive_link&ouid=118228466349625254909&rtpof=true&sd=true)

[Data S9. KEGG result](https://docs.google.com/spreadsheets/d/1nbdRmL-CRF3mlQ9FweQmIN9Ir2KKTJns/edit?usp=drive_link&ouid=118228466349625254909&rtpof=true&sd=true)
