# Supplementary material for: A Panel of Circulating Exosomal sncRNAs Associated With Lung Cancer Risk up to 10 Years in Advance
Source: Adv Sci (Weinh). 2026 Aug 14;13(57):e76729. doi: 10.1002/advs.76729 (PMC13474057; doi:10.1002/advs.76729)
Supplement: Supplementary file 2 [file ADVS-13-e76729-s002.zip › Data S1_CHTN sample info.pdf]

batch	ID	Sex	Age	Race	Diagnosis
CHTN	sample001	Male	52.15	White	cancer
CHTN	sample002	Male	64.39	White	cancer
CHTN	sample003	Female	56.56	Black	cancer
CHTN	sample004	Male	58.81	White	cancer
CHTN	sample005	Male	69.02	White	cancer
CHTN	sample006	Male	68.38	White	cancer
CHTN	sample007	Female	58.16	White	cancer
CHTN	sample008	Male	58.88	White	cancer
CHTN	sample010	Male	64.33	White	cancer
CHTN	sample011	Male	64.48	White	cancer
CHTN	sample012	Male	69.5	White	cancer
CHTN	sample013	Female	60.45	White	cancer
CHTN	sample014	Male	57.78	White	cancer
CHTN	sample015	Male	69.54	White	cancer
CHTN	sample016	Female	58.07	White	cancer
CHTN	sample017	Male	63.92	White	cancer
CHTN	sample018	Male	60.88	White	cancer
CHTN	sample019	Male	57.48	White	cancer
CHTN	sample020	Male	49.93	White	cancer
CHTN	sample021	Male	62.05	Black	cancer
CHTN	sample022	Female	66.16	White	cancer
CHTN	sample023	Male	71.71	Black	cancer
CHTN	sample024	Female	60.07	White	cancer
CHTN	sample025	Male	69.3	White	cancer
CHTN	sample026	Male	61.27	White	cancer
CHTN	sample027	Female	59.86	White	cancer
CHTN	sample028	Male	56.84	White	cancer
CHTN	sample029	Male	55.52	White	cancer
CHTN	sample030	Female	66.07	Black	cancer
CHTN	sample031	Female	68.75	White	cancer
CHTN	sample032	Male	64.06	Black	cancer
CHTN	sample033	Female	65.82	White	cancer
CHTN	sample034	Female	59.2	White	cancer
CHTN	sample035	Male	60.65	White	cancer
CHTN	sample036	Female	70.32	White	cancer
CHTN	sample037	Female	64	White	cancer
CHTN	sample038	Female	54.19	White	cancer
CHTN	sample039	Female	73.1	White	cancer
CHTN	sample040	Female	67.11	White	cancer
CHTN	sample041	Female	68.84	White	cancer
CHTN	sample044	Female	65.49	White	cancer
CHTN	sample045	Male	65.55	White	cancer
CHTN	sample046	Female	54.79	White	cancer

CHTN	sample047	Male	62.08	Black	cancer
CHTN	sample048	Male	63.48	White	cancer
CHTN	sample049	Male	54.6	White	cancer
CHTN	sample050	Female	47.12	Black	cancer
CHTN	sample051	Female	49.93	White	cancer
CHTN	sample052	Male	64.94	White	cancer
CHTN	sample053	Female	73.32	White	cancer
CHTN	sample054	Female	61.2	White	cancer
CHTN	sample055	Male	60.28	Black	cancer
CHTN	sample056	Male	61.06	White	cancer
CHTN	sample057	Male	63.92	White	cancer
CHTN	sample058	Male	63.67	Black	cancer
CHTN	sample059	Female	53.12	White	cancer
CHTN	sample060	Male	69.44	White	cancer
CHTN	sample061	Female	54.43	White	cancer
CHTN	sample062	Female	54.52	White	cancer
CHTN	sample063	Female	63.81	White	cancer
CHTN	sample064	Female	62.34	White	cancer
CHTN	sample065	Female	58.54	White	cancer
CHTN	sample066	Male	64.61	White	cancer
CHTN	sample067	Male	57.69	White	cancer
CHTN	sample068	Female	57.34	White	cancer
CHTN	sample069	Male	65.64	White	cancer
CHTN	sample070	Male	63.23	White	cancer
CHTN	sample071	Male	63.78	White	cancer
CHTN	sample072	Female	70.52	White	cancer
CHTN	sample073	Male	60.54	White	cancer
CHTN	sample074	Female	58.51	Black	benign
CHTN	sample075	Female	63.52	White	benign
CHTN	sample077	Female	55.65	White	benign
CHTN	sample078	Male	60.7	White	benign
CHTN	sample079	Male	64.21	White	benign
CHTN	sample080	Female	63.73	White	benign
CHTN	sample081	Female	67.42	White	benign
CHTN	sample082	Female	65.08	Black	benign
CHTN	sample083	Male	61.57	White	benign
CHTN	sample084	Female	69.02	White	benign
CHTN	sample085	Male	68.33	Black	benign
CHTN	sample086	Female	64.56	White	benign
CHTN	sample087	Female	60.83	White	benign
CHTN	sample088	Male	67	White	benign
CHTN	sample089	Male	58.57	Black	benign
CHTN	sample090	Male	62.53	White	benign
CHTN	sample091	Female	58.7	White	benign

CHTN	sample092	Male	59.9	White	benign
CHTN	sample093	Female	66.59	White	benign
CHTN	sample094	Female	55.6	White	benign
CHTN	sample095	Female	64.14	White	benign
CHTN	sample096	Male	66.57	White	benign
CHTN	sample097	Female	66.87	White	benign
CHTN	sample098	Female	62.02	White	benign
CHTN	sample099	Male	58.36	White	benign
CHTN	sample100	Female	58.15	White	benign
CHTN	sample101	Male	66.75	White	benign
CHTN	sample102	Male	56.31	White	benign
CHTN	sample103	Female	62.91	White	benign
CHTN	sample104	Female	62.91	White	benign
CHTN	sample105	Male	58.35	White	benign
CHTN	sample106	Male	63.47	Black	benign
CHTN	sample107	Male	55.15	White	benign
CHTN	sample108	Male	58.73	Black	benign
CHTN	sample109	Male	63.46	White	benign
CHTN	sample110	Male	49.09	White	benign
CHTN	sample111	Male	58.08	White	benign
CHTN	sample112	Male	56.39	White	benign
CHTN	sample113	Male	58.96	White	benign
CHTN	sample114	Female	58.75	White	benign
CHTN	sample115	Female	65.87	White	benign
CHTN	sample116	Female	66.76	White	benign
CHTN	sample117	Male	64.67	White	benign
CHTN	sample118	Female	49.64	White	benign
CHTN	sample119	Male	62.84	White	benign
CHTN	sample120	Male	64.97	White	benign
CHTN	sample121	Male	62.76	White	benign
CHTN	sample122	Female	47.67	Black	benign
CHTN	sample123	Female	57.13	White	benign
CHTN	sample124	Female	62.69	Black	benign
CHTN	sample125	Female	65.6	Black	benign
CHTN	sample126	Female	62.37	White	benign
CHTN	sample127	Female	63.15	White	benign
CHTN	sample128	Male	61.41	White	benign
CHTN	sample129	Female	63.99	Black	benign
CHTN	sample130	Male	58.05	White	benign
CHTN	sample131	Male	60.21	White	benign
CHTN	sample132	Female	65.86	White	benign
CHTN	sample133	Female	57.14	White	benign
CHTN	sample134	Male	60.82	White	benign
CHTN	sample135	Female	58.44	White	benign

CHTN	sample136	Male	69.49	White	benign
CHTN	sample137	Female	66.04	White	benign
CHTN	sample138	Female	61.39	White	benign
CHTN	sample139	Male	55.58	White	benign
CHTN	sample140	Female	70.33	White	benign
CHTN	sample141	Female	68.32	White	benign
CHTN	sample142	Female	67.63	White	benign
CHTN	sample143	Female	59.56	White	benign
CHTN	sample144	Female	59.12	White	benign
CHTN	sample145	Male	55.44	White	benign
CHTN	sample146	Male	55.14	White	benign
CHTN	sample147	Male	65.35	White	benign
CHTN	sample148	Male	58.76	Black	benign
CHTN	sample149	Female	65.62	White	benign
CHTN	sample150	Male	66.2	Black	benign
CHTN	sample151	Male	63.14	Black	benign
CHTN	sample152	Male	68.75	White	benign
CHTN	sample153	Female	21	Black	normal
CHTN	sample154	Female	21	Black	normal
CHTN	sample155	Female	34	White	normal
CHTN	sample156	Male	42	White	normal
CHTN	sample157	Female	57	Black	normal
CHTN	sample158	Female	58	White	normal
CHTN	sample159	Female	66	White	normal
CHTN	sample160	Female	69	White	normal
CHTN	sample162	Female	48	White	normal
CHTN	sample163	Female	71	White	normal
CHTN	sample164	Male	76	White	normal
CHTN	sample167	Female	56	White	normal
CHTN	sample168	Male	51	Black	normal
CHTN	sample169	Male	68	White	normal
CHTN	sample170	Male	61	Black	normal
CHTN	sample172	Male	62	White	normal
CHTN	sample173	Male	59	White	normal
CHTN	sample174	Male	59	White	normal
CHTN	sample175	Male	52	White	normal
CHTN	sample176	Male	55	White	normal
CHTN	sample177	Female	58	White	normal
CHTN	sample178	Male	53	Black	normal
CHTN	sample179	Male	61	White	normal
CHTN	sample180	Male	59	Black	normal
CHTN	sample181	male	74	White	normal
CHTN	sample182	male	69	Black	normal
CHTN	sample183	male	68	White	normal

CHTN	sample184	male	69	White	normal
CHTN	sample185	male	75	White	normal
CHTN	sample186	male	73	White	normal
CHTN	sample187	female	65	White	normal
CHTN	sample188	female	62	White	normal
CHTN	sample189	female	66	White	normal
CHTN	sample190	male	69	White	normal
CHTN	sample191	female	63	Black	normal
CHTN	sample192	female	70	Black	normal
CHTN	sample193	female	66	White	normal
CHTN	sample194	female	68	White	normal
