# Supplementary material for: A Panel of Circulating Exosomal sncRNAs Associated With Lung Cancer Risk up to 10 Years in Advance
Source: Adv Sci (Weinh). 2026 Aug 14;13(57):e76729. doi: 10.1002/advs.76729 (PMC13474057; doi:10.1002/advs.76729)
Supplement: Supplementary file 2 [file ADVS-13-e76729-s002.zip › Data S3_landmark analysis ROC-AUC.pdf]

fold	window	w_start	w_end	w_mid	n_case	n_ctrl	auc
Fold 1	0-5y	0	5	2.5	18	130	0.955
Fold 1	5-10y	5	10	7.5	22	115	0.935
Fold 1	10-15y	10	15	12.5	27	29	0.947
Fold 2	0-5y	0	5	2.5	18	130	0.951
Fold 2	5-10y	5	10	7.5	22	115	0.938
Fold 2	10-15y	10	15	12.5	27	29	0.918
Fold 3	0-5y	0	5	2.5	18	130	0.943
Fold 3	5-10y	5	10	7.5	22	115	0.92
Fold 3	10-15y	10	15	12.5	27	29	0.93
Fold 4	0-5y	0	5	2.5	18	130	0.96
Fold 4	5-10y	5	10	7.5	22	115	0.927
Fold 4	10-15y	10	15	12.5	27	29	0.952
Fold 5	0-5y	0	5	2.5	18	130	0.957
Fold 5	5-10y	5	10	7.5	22	115	0.942
Fold 5	10-15y	10	15	12.5	27	29	0.957
