# Supplementary material for: A Panel of Circulating Exosomal sncRNAs Associated With Lung Cancer Risk up to 10 Years in Advance
Source: Adv Sci (Weinh). 2026 Aug 14;13(57):e76729. doi: 10.1002/advs.76729 (PMC13474057; doi:10.1002/advs.76729)
Supplement: Supplementary file 2 [file ADVS-13-e76729-s002.zip › Data S4_landmark analysis PRC-AUPRC.pdf]

fold	window	window_or	w_start	w_end	w_mid	n_case	n_ctrl	auprc	baseline
Fold 1	0-5y	(0,5]	0	5	2.5	18	130	0.726	0.122
Fold 1	5-10y	(5,10]	5	10	7.5	22	115	0.674	0.161
Fold 1	10-15y	(10,15]	10	15	12.5	27	29	0.327	0.482
Fold 2	0-5y	(0,5]	0	5	2.5	18	130	0.727	0.122
Fold 2	5-10y	(5,10]	5	10	7.5	22	115	0.665	0.161
Fold 2	10-15y	(10,15]	10	15	12.5	27	29	0.332	0.482
Fold 3	0-5y	(0,5]	0	5	2.5	18	130	0.733	0.122
Fold 3	5-10y	(5,10]	5	10	7.5	22	115	0.673	0.161
Fold 3	10-15y	(10,15]	10	15	12.5	27	29	0.33	0.482
Fold 4	0-5y	(0,5]	0	5	2.5	18	130	0.723	0.122
Fold 4	5-10y	(5,10]	5	10	7.5	22	115	0.67	0.161
Fold 4	10-15y	(10,15]	10	15	12.5	27	29	0.326	0.482
Fold 5	0-5y	(0,5]	0	5	2.5	18	130	0.726	0.122
Fold 5	5-10y	(5,10]	5	10	7.5	22	115	0.664	0.161
Fold 5	10-15y	(10,15]	10	15	12.5	27	29	0.325	0.482
